# TDP-43 loss induces cryptic polyadenylation in ALS/FTD

**DOI:** 10.1038/s41593-025-02050-w

**Published:** 2025-10-21

**Authors:** Sam Bryce-Smith, Anna-Leigh Brown, Max Z. Y. J. Chien, Dario Dattilo, Puja R. Mehta, Francesca Mattedi, Simone Barattucci, Alla Mikheenko, Matteo Zanovello, Flaminia Pellegrini, Sara Emad El-Agamy, Matthew Yome, Sarah E. Kargbo-Hill, Yue A. Qi, Kai Sun, Eugeni Ryadnov, Yixuan Wan, NYGC ALS Consortium, Hemali Phatnani, Justin Kwan, Dhruv Sareen, James R. Broach, Zachary Simmons, Ximena Arcila-Londono, Edward B. Lee, Vivianna M. Van Deerlin, Neil A. Shneider, Ernest Fraenkel, Lyle W. Ostrow, Frank Baas, Noah Zaitlen, James D. Berry, Andrea Malaspina, Gregory A. Cox, Leslie M. Thompson, Steve Finkbeiner, Efthimios Dardiotis, Timothy M. Miller, Siddharthan Chandran, Suvankar Pal, Eran Hornstein, Daniel J. MacGowan, Terry Heiman-Patterson, Molly G. Hammell, Nikolaos. A. Patsopoulos, Joshua Dubnau, Avindra Nath, Robert Bowser, Matthew Harms, Eleonora Aronica, Mary Poss, Jennifer Phillips-Cremins, John Crary, Nazem Atassi, Dale J. Lange, Darius J. Adams, Leonidas Stefanis, Marc Gotkine, Robert H. Baloh, Suma Babu, Sabrina Paganoni, Ophir Shalem, Colin Smith, Bin Zhang, Justin Kwan, Thomas Blanchard, Brent Harris, Iris Broce, Vivian Drory, John Ravits, Corey McMillan, Vilas Menon, Lani Wu, Steven Altschuler, Yossef Lerner, Rita Sattler, Kendall Van Keuren-Jensen, Orit Rozenblatt-Rosen, Kerstin Lindblad-Toh, Katharine Nicholson, Peter Gregersen, Jeong-Ho Lee, Oleg Butovsky, Matt Brauer, Tara Nickerson, Shameek Biswas, Kimberly A. Wilson, Sulev Koks, Stephen Muljo, Bryan J. Traynor, Robert Moccia, Seng Cheng, Andrew Deubler, Giovanni Coppola, Mickey Atwal, Michael Cantor, William Salerno, Eli Stahl, Matt Anderson, David Frendewey, Daphne Koller, Mary Rozenman, Towfique Raj, Pietro Fratta, Jose Norberto S. Vargas, Nicol Birsa, Towfique Raj, Jack Humphrey, Matthew Keuss, Oscar G. Wilkins, Michael Ward, Maria Secrier, Pietro Fratta

**Affiliations:** 1 UCL Queen Square Motor Neuron Disease Centre, Department of Neuromuscular Diseases, UCL Queen Square Institute of Neurology, University College London, London, UK; 2 The Francis Crick Institute, London, UK; 3 National Institute of Neurological Disorders and Stroke, National Institutes of Health, Bethesda, MD USA; 4 Nash Family Department of Neuroscience & Friedman Brain Institute, Icahn School of Medicine at Mount Sinai, New York, NY USA; 5 Ronald M. Loeb Center for Alzheimer’s Disease, Icahn School of Medicine at Mount Sinai, New York, NY USA; 6 Department of Genetics and Genomic Sciences & Icahn Institute for Data Science and Genomic Technology, Icahn School of Medicine at Mount Sinai, New York, NY USA; 7 Estelle and Daniel Maggin Department of Neurology, Icahn School of Medicine at Mount Sinai, New York, NY USA; 8 UCL Genetics Institute, Department of Genetics, Evolution and Environment, University College London, London, UK; 9 Center for Genomics of Neurodegenerative Disease (CGND), New York Genome Center, New York, NY USA; 10 Department of Neurology, Lewis Katz School of Medicine, Temple University, Philadelphia, PA USA; 11 Cedars-Sinai Biomanufacturing Center, Department of Biomedical Sciences, Board of Governors Regenerative Medicine Institute and Brain Program, Cedars-Sinai Medical Center, Los Angeles, CA USA; 12 Department of Biochemistry and Molecular Biology, Penn State Institute for Personalized Medicine, The Pennsylvania State University, Hershey, PA USA; 13 Department of Neurology, The Pennsylvania State University, Hershey, PA USA; 14 Department of Neurology, Henry Ford Health System, Detroit, MI USA; 15 Department of Pathology and Laboratory Medicine, Perelman School of Medicine, University of Pennsylvania, Philadelphia, PA USA; 16 Department of Neurology, Center for Motor Neuron Biology and Disease, Institute for Genomic Medicine, Columbia University, New York, NY USA; 17 Department of Biological Engineering, Massachusetts Institute of Technology, Cambridge, MA USA; 18 Department of Neurology, Johns Hopkins School of Medicine, Baltimore, MD USA; 19 Department of Neurogenetics, Academic Medical Centre, Amsterdam and Leiden University Medical Center, Leiden, The Netherlands; 20 Department of Medicine, Lung Biology Center, University of California, San Francisco, San Francisco, CA USA; 21 ALS Multidisciplinary Clinic, Neuromuscular Division, Department of Neurology, Harvard Medical School, and Neurological Clinical Research Institute, Massachusetts General Hospital, Boston, MA USA; 22 Centre for Neuroscience and Trauma, Blizard Institute, Barts and The London School of Medicine and Dentistry, Queen Mary University of London, London, UK; 23 Department of Neurology, Basildon University Hospital, Basildon, UK; 24 The Jackson Laboratory, Bar Harbor, ME USA; 25 Department of Psychiatry & Human Behavior, Department of Biological Chemistry, School of Medicine, and Department of Neurobiology and Behavior, School of Biological Sciences, University California, Irvine, Irvine, CA USA; 26 Taube/Koret Center for Neurodegenerative Disease Research, Roddenberry Center for Stem Cell Biology and Medicine, Gladstone Institute, San Francisco, CA USA; 27 Department of Neurology & Sensory Organs, University of Thessaly, Thessaly, Greece; 28 Department of Neurology, Washington University in St. Louis, St. Louis, MO USA; 29 Centre for Clinical Brain Sciences, Anne Rowling Regenerative Neurology Clinic, Euan MacDonald Centre for Motor Neurone Disease Research, University of Edinburgh, Edinburgh, UK; 30 Department of Molecular Genetics, Weizmann Institute of Science, Rehovot, Israel; 31 Department of Neurology, Icahn School of Medicine at Mount Sinai, New York, NY USA; 32 Center for Neurodegenerative Disorders, Department of Neurology, the Lewis Katz School of Medicine, Temple University, Philadelphia, PA USA; 33 Cold Spring Harbor Laboratory, Cold Spring Harbor, NY USA; 34 Computer Science and Systems Biology Program, Ann Romney Center for Neurological Diseases, Department of Neurology and Division of Genetics in Department of Medicine, Brigham and Women’s Hospital, Boston, MA USA; 35 Harvard Medical School, Boston, MA USA; 36 Program in Medical and Population Genetics, Broad Institute, Cambridge, MA USA; 37 Department of Anesthesiology, Stony Brook University, Stony Brook, NY USA; 38 Section of Infections of the Nervous System, National Institute of Neurological Disorders and Stroke, National Institutes of Health, Bethesda, MD USA; 39 Department of Neurology, Barrow Neurological Institute, St. Joseph’s Hospital and Medical Center, Department of Neurobiology, Barrow Neurological Institute, St. Joseph’s Hospital and Medical Center, Phoenix, AZ USA; 40 Department of Neurology, Division of Neuromuscular Medicine, Columbia University, New York, NY USA; 41 Department of Neuropathology, Academic Medical Center, University of Amsterdam, Amsterdam, The Netherlands; 42 Department of Biology and Veterinary and Biomedical Sciences, The Pennsylvania State University, University Park, PA USA; 43 New York Stem Cell Foundation, Department of Bioengineering, School of Engineering and Applied Sciences, University of Pennsylvania, Philadelphia, PA USA; 44 Department of Pathology, Fishberg Department of Neuroscience, Friedman Brain Institute, Ronald M. Loeb Center for Alzheimer’s Disease, Icahn School of Medicine at Mount Sinai, New York, NY USA; 45 Department of Neurology, Harvard Medical School, Neurological Clinical Research Institute, Massachusetts General Hospital, Boston, MA USA; 46 Department of Neurology, Hospital for Special Surgery and Weill Cornell Medical Center, New York, NY USA; 47 Medical Genetics, Atlantic Health System, Morristown Medical Center, Morristown, NJ USA; 48 Overlook Medical Center, Summit, NJ USA; 49 Center of Clinical Research, Experimental Surgery and Translational Research, Biomedical Research Foundation of the Academy of Athens (BRFAA), Athens, Greece; 50 1st Department of Neurology, Eginition Hospital, Medical School, National and Kapodistrian University of Athens, Athens, Greece; 51 Neuromuscular/EMG Service and ALS/Motor Neuron Disease Clinic, Hebrew University-Hadassah Medical Center, Jerusalem, Israel; 52 Board of Governors Regenerative Medicine Institute, Los Angeles, CA USA; 53 Department of Neurology, Cedars-Sinai Medical Center, Los Angeles, CA USA; 54 Neurological Clinical Research Institute, Massachusetts General Hospital, Boston, MA USA; 55 Harvard Medical School, Department of Physical Medicine & Rehabilitation, Spaulding Rehabilitation Hospital, Boston, MA USA; 56 Center for Cellular and Molecular Therapeutics, Children’s Hospital of Philadelphia, Philadelphia, PA USA; 57 Department of Genetics, Perelman School of Medicine, University of Pennsylvania, Philadelphia, PA USA; 58 Centre for Clinical Brain Sciences, University of Edinburgh, Edinburgh, UK; 59 Euan MacDonald Centre for Motor Neurone Disease Research, University of Edinburgh, Edinburgh, UK; 60 Department of Genetics and Genomic Sciences, Icahn Institute of Data Science and Genomic Technology, Icahn School of Medicine at Mount Sinai, New York, NY USA; 61 University of Maryland Brain and Tissue Bank and NIH NeuroBioBank, Baltimore, MD USA; 62 Department of Neuropathology, Georgetown Brain Bank, Georgetown Lombardi Comprehensive Cancer Center, Georgetown University Medical Center, Washington, DC USA; 63 Neuroradiology Section, Department of Radiology and Biomedical Imaging, University of California, San Francisco, San Francisco, CA USA; 64 Neuromuscular Diseases Unit, Department of Neurology, Tel Aviv Sourasky Medical Center, Sackler Faculty of Medicine, Tel Aviv University, Tel Aviv, Israel; 65 Department of Neuroscience, University of California, San Diego, La Jolla, CA USA; 66 Department of Neurology, University of Pennsylvania Perelman School of Medicine, Philadelphia, PA USA; 67 Department of Neurology, Columbia University Medical Center, New York, NY USA; 68 Department of Pharmaceutical Chemistry, University of California, San Francisco, San Francisco, CA USA; 69 Hadassah Hebrew University, Jerusalem, Israel; 70 Department of Translational Neuroscience, Barrow Neurological Institute, Phoenix, AZ USA; 71 The Translational Genomics Research Institute (TGen), Phoenix, AZ USA; 72 Broad Institute, Cambridge, MA USA; 73 Massachusetts General Hospital, Boston, MA USA; 74 Institute of Molecular Medicine, Feinstein Institutes for Medical Research, Northwell Health, Manhasset, NY USA; 75 Korea Advanced Institute of Science and Technology (KAIST), Daejeon, South Korea; 76 Ann Romney Center for Neurologic Diseases, Brigham and Women’s Hospital, Harvard Medical School, Boston, MA USA; 77 Maze Therapeutics, South San Francisco, CA USA; 78 Bristol Myers Squibb, New York, NY USA; 79 Perron Institute for Neurological and Translational Science, Nedlands, Western Australia Australia; 80 Integrative Immunobiology Section, National Institute of Allergy and Infectious Disease, National Institutes of Health, Bethesda, MD USA; 81 Neuromuscular Disease Research Section, National Institute of Aging, Bethesda, MD USA; 82 Pfizer, New York, NY USA; 83 Regeneron, Tarrytown, NY USA; 84 Insitro, South San Francisco, CA USA

**Keywords:** Cellular neuroscience, Amyotrophic lateral sclerosis, RNA splicing, Computational biology and bioinformatics

## Abstract

Nuclear depletion and cytoplasmic aggregation of the RNA-binding protein TDP-43 are cellular hallmarks of amyotrophic lateral sclerosis (ALS). TDP-43 nuclear loss causes de-repression of cryptic exons, yet cryptic alternative polyadenylation (APA) events have been largely overlooked. In this study, we developed a bioinformatic pipeline to reliably identify alternative last exons, 3’ untranslated region (3’UTR) extensions and intronic polyadenylation APA event types, and we identified cryptic APA sites induced by TDP-43 loss in induced pluripotent stem cell (iPSC)-derived neurons. TDP-43 binding sites are enriched at sites of these cryptic events, and TDP-43 can both repress and enhance APA. All categories of cryptic APA were also identified in ALS and frontotemporal dementia (FTD) postmortem brain tissue. RNA sequencing (RNA-seq), thiol(SH)-linked alkylation for the metabolic sequencing of RNA (SLAM-seq) and ribosome profiling (Ribo-seq) revealed that distinct cryptic APA categories have different downstream effects on transcript levels and that cryptic 3’UTR extensions can increase RNA stability, leading to increased translation. In summary, we demonstrate that TDP-43 nuclear depletion induces cryptic APA, expanding the palette of known consequences of TDP-43.

## Abstract (web summary)

The authors find that TDP-43 loss of function—the pathology defining the neurodegenerative conditions ALS and FTD—induces novel mRNA polyadenylation events, which have different effects, including an increase in RNA stability, leading to higher protein levels.

## Main

Cytoplasmic aggregates and nuclear depletion of TDP-43 are pathological hallmarks of a spectrum of neurodegenerative diseases, including over 97% of ALS cases^1^, 45% of FTD cases^2^ and over 50% of Alzheimer’s disease cases^3^. Under normal conditions, TDP-43 is a predominantly nuclear protein with multiple roles in regulation of RNA processing and metabolism, including alternative splicing, APA^4–6^ and transport^7^. Considerable attention has been drawn to the ability of TDP-43 to repress the inclusion of pre-mRNA sequences in mature transcripts^8^: loss of nuclear TDP-43 leads to the inclusion of ‘cryptic’ exons both in vitro and in postmortem tissue^9^, contributing to disease progression^10,11^. Cryptic exons can lead to protein loss through RNA degradation by nonsense-mediated decay^12^ or can be translated to produce cryptic peptides^13,14^.

Cleavage and polyadenylation defines the 3′ end of last exons and subsequently mature transcripts^15^. Up to 70% of human protein-coding and long non-coding RNA (lncRNA) genes can undergo polyadenylation at multiple locations in the gene body (APA) and can be subdivided into three main categories of events: alternative last exons (ALEs), 3’UTR extensions (3’Ext) and ‘composite’ intronic polyadenylation (IPA) events. In ALEs, the poly(A) usage is determined by an upstream alternative splice junction, which defines an alternative last exon. In 3’Ext events, APA sites are independent of splice junctions and occur downstream of annotated distal 3’UTRs to affect 3’UTR sequence and length, which is implicated in the regulation of transcript stability, localization and translation^16^. Finally, in IPA events, APA occurs within introns in the absence of upstream alternative splicing, giving rise to transcripts with different protein-coding potential and can affect full-length protein dosage^17,18^.

TDP-43-regulated cryptic APA has not been systematically explored in a neuronal context. Here we report widespread cryptic APA upon TDP-43 depletion in cell models, including 3’Ext and IPA events that were not previously detected with conventional splicing analyses. A substantial number is expressed in postmortem ALS and ALS/FTD tissue with TDP-43 loss, underlining their potential involvement in pathogenic mechanisms and/or utility as biomarkers of TDP-43 pathology. We focus on a novel class of 3’Ext APA and use metabolic labeling to demonstrate that such cryptic 3’Ext is associated with increased RNA stability, can localize to the cytoplasm and is translated, leading to an increase in protein levels.

Our data, therefore, identify a novel consequence for cryptic RNA processing and show that, in addition to leading to protein reduction or the formation of altered proteins, this can also lead to overexpression of normal proteins and an increase in their function.

## Results

### Identification of cryptic APA events induced by TDP-43 loss

**Fig. 1:**
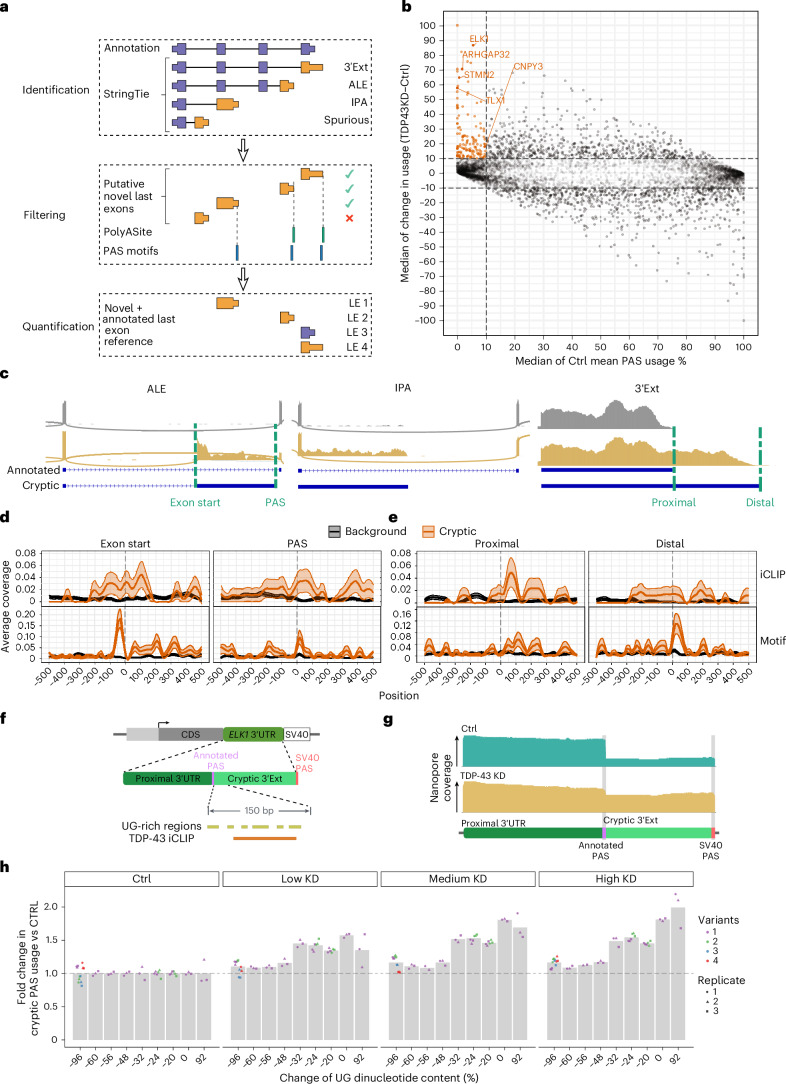
TDP-43 depletion induces cryptic APA in a compendium of in vitro TDP-43 datasets. **a**, Computational pipeline inferring differential last exon (LE) usage from bulk RNA-seq. Putative novel last exons (orange) are identified by comparing StringTie^19^ assembled transcripts (condition mean TPM > 1) to reference transcripts (purple). Putative last exons with a PAS <100 nt from PolyASite^20^ PAS or containing a conserved poly(A) signal hexamer^21^ (final 100 nt) are quantified with annotated last exons using Salmon^22^ and assessed for differential usage using DEXSeq^23^. **b**, APA upon TDP-43 knockdown (TDP43KD). Points: PAS with adjusted *P* < 0.05 in ≥1 dataset (median values when >1 dataset). Cryptic PAS (orange): adjusted *P* < 0.05, mean control (Ctrl) usage <10% and TDP43KD–CTRL usage >10%. **c**, Cryptic APA RNA-seq coverage traces in control (gray) and TDP-43 knockdown (gold) i3Neuron. ALE: *ARHGAP32*. IPA: *ANKRD27*. 3’Ext: *TLX1*. Dashed lines: landmarks assessed for TDP-43 binding (**d**,**e**). All events are visualized in sense orientation. **d**, TDP-43 binding around ALE boundaries. Exon start: first nucleotide of the last exon. Top, mean SH-SY5Y TDP-43 iCLIP peak coverage (*n* = 2) ±1 s.e.m. (shaded interval) of positions relative to landmarks in cryptic (orange, *n* = 92) versus background (black, *n* = 929) ALEs. Two-sided Fisher’s exact test in the plotting window (exon start *P* = 0.005, PAS *P* = 0.019). Bottom, mean YG-containing hexamer coverage (Supplementary Fig. 3a) ±1 s.e.m. (shaded interval). **e**, TDP-43 binding maps around 3’Ext alternative PAS. Top, as in **d** (top) for cryptic (orange, *n* = 86) and background (black, *n* = 798) 3’Exts. Proximal *P* = 0.031, distal *P* = 0.003. Bottom, as in **d** (bottom) for **e** (top). **f**, *ELK1* fluorescent reporter. CDS: mGreenLantern coding sequence. *ELK1* 3’UTR, proximal 3’UTR and the first 800 bp of cryptic 3’Ext. SV40, *SV40* PAS. **g**, Nanopore sequencing traces of the reporter in TDP-43 knockdown SK-N-BE(2) cells. **h**, Reporter distal PAS usage upon increasing TDP-43 knockdown (low: 30, medium: 60, high: 1,000 ng ml^−1^ doxycycline). Bars denote mean PAS usage fold change versus controls. *n* = 3 per variant. −96%: four variants; −20% and −24%: two variants; remaining: one variant.

Although the role of TDP-43 in regulating APA and cryptic splicing is well known, cryptic APA occurring upon TDP-43 loss of function has yet to be explored. To comprehensively address this question, we curated a compendium of publicly available and newly generated bulk RNA-seq datasets with TDP-43 depletion (Supplementary Table 1). We assembled a computational pipeline to identify novel last exons from RNA-seq data, which defines last exon frames using StringTie^19^ and then filters and categorizes as spurious predicted 3’ ends lacking the presence of reference poly(A) sites^20^ or a conserved poly(A) signal hexamer^21^ (Fig. 1a). Isoform-level quantification was performed using Salmon^22^, and differential usage between experimental conditions was assessed using DEXSeq^23^.

This approach allowed us to subdivide our events into three main categories—ALEs, IPAs and 3’Ext (Fig. 1a)—overcoming the limitations of comparable available tools that focus on specific event categories^24–28^. APA events were widespread, and we defined cryptic APA events as ones with less than 10% mean usage in controls and more than 10% usage change after TDP-43 knockdown. We identified 227 cryptic APAs to be present in at least one dataset (adjusted *P* < 0.05; Fig. 1b, Supplementary Fig. 1 and Supplementary Table 2). Cryptic ALEs (*n* = 92) included previously identified cryptic exons such as *STMN2*, *ARHGAP32* and *RSF1* (Fig. 1b and Supplementary Fig. 2). In total, 108 3’UTR cryptics were identified, of which 86 are novel 3’UTR extensions (3’Ext; for example, *TLX1*; Fig. 1c), and 20 were 3’UTR shortening events at loci with novel 3’Exts (3’shortening). Twenty IPA events were also detected, including *CNPY3*, which was identified with an independent bioinformatics approach and experimentally validated^29^. The remaining nine events could not be uniquely assigned to ALEs or IPAs based on annotation and are defined as ‘complex’. Multiple non-cryptic APA events were also detected and are reported in Supplementary Table 3.

**Extended Data Fig. 1:**
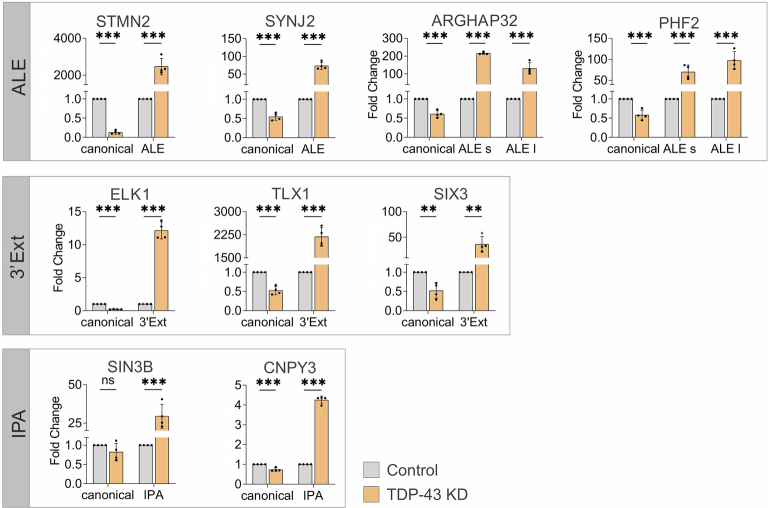
3’ RACE validation of cryptic APAs in i3Neurons. RT-qPCR analysis after 3’RACE for the indicated 3’UTRs upon TDP-43 depletion (“TDP-43 KD”) in i3Neurons. The RNA expression levels were normalized against GAPDH mRNA and expressed as relative fold change with respect to the control condition (“Control”) set to a value of 1. Data are represented as the mean of the fold change ± standard deviation. n=4 biological replicates. Statistical analyses were performed using two-sided, Student unpaired t-test (n.s. p>0.05, * p<0.05, ** p<0.01, **** p<0.0001). ALE: alternative last exon (s short, l long), 3’Ext: 3’UTR extension, IPA: intronic polyadenylation. Exact p-values are reported in the form (canonical, ALE/IPA/3’Ext/ALE short, ALE long). STMN2 (p = 2.804×10^−8^, 3.280×10^−5^). SYNJ2 (p = 7.577×10^−5^, 6.916×10^−6^). ARGHAP32 (p = 1.536×10^−4^, 6.094×10^−10^, 2.018×10^−4^). PHF2 (p = 6.727×10^−4^, 1.680×10^−4^, 9.785×10^−5^). ELK1 (p = 8.711×10^−10^, 2.295×10^−6^). TLX1 (p = 1.271×10^−4^, 6.779×10^−6^). SIX3 (p = 3.248×10^−3^, 3.711×10^−3^). SIN3B (p = 1.735×10^−1^, 3.490×10^−4^). CNPY3 (p = 8.970×10^−4^, 5.032×10^−8^). Source data

We experimentally validated strong activation of cryptic APA and confirmed the expression of multiple predicted PASs by performing 3’ rapid amplification of cDNA ends (3’RACE) in i3Neurons (Extended Data Fig. 1) and inspecting poly(A)-tail ligation-dependent, oligo-dT primer-free i3Neuron direct RNA nanopore sequencing^13^ (Supplementary Fig. 3a−c). We further evaluated global cryptic polyadenylation site (PAS) precision by pooling across TDP-43 depletion RNA-seq samples poly(A)-tail-containing reads (PATRs; Supplementary Fig. 3d), which allows independent defining of PASs^30^. Cryptic and expression-matched annotated PASs were similarly identified, further supporting the novel cryptic APA events (Supplementary Fig. 3e). Finally, the commonly used tool DaPars2 (ref. ^31^), when provided with the predicted 3’Ext coordinates, reproduced cryptic 3’Ext activation (Supplementary Fig. 4). These findings collectively support the validity of our cryptic APA discovery pipeline.

Out of 227 cryptic APAs detected by our analysis across datasets, most (138) satisfied cryptic expression criteria (<10% mean usage in controls and >10% usage change after TDP-43 knockdown) when considering the median across datasets. Fifty-one APAs were, instead, consistently below 10% usage threshold in controls but did not sufficiently increase after TDP-43 depletion to meet the cryptic criteria definition across datasets. Twenty-eight APAs showed, instead, a significant increase upon TDP-43 loss across datasets but had more than 10% median usage in controls, therefore placing them outside the cryptic criteria but demonstrating consistent regulation by TDP-43 (Supplementary Fig. 5). Altogether, these data highlight a widespread presence of cryptic APA upon TDP-43 loss.

### TDP-43 binding both represses and enhances poly(A) site choice

Next, we investigated TDP-43 binding patterns around cryptic APAs using TDP-43 individual-nucleotide resolution UV crosslinking and immunoprecipitation (iCLIP) data generated in SH-SY5Y cells^10^. We focused on ALEs and 3’Ext events as the low number of IPA and 3’shortening events (*n* = 20 in both cases) did not allow reliable binding profile inferences. TDP-43 binding was enriched around the splice acceptor of cryptic ALEs, as previously described in cryptic splice junctions, and downstream of the cryptic PAS of ALEs (Fig. 1d), supporting TDP-43 acting as a repressor of both splicing and polyadenylation. Intriguingly, TDP-43 binding was also enriched immediately downstream of the annotated proximal PAS of 3’Ext events (Fig. 1e), supporting a role for TDP-43 in enhancing poly(A) usage, consistent with previous reports of TDP-43 binding with respect to regulated PAS^5^.

iCLIP data, typically generated in control cells, are not sensitive in detecting binding to cryptic 3’Ext regions, as these events can be detected only at very low levels with physiological TDP-43 presence. We, therefore, sought to corroborate our findings by adapting PEKA^32^ to infer de novo hexamer enrichment relative to cryptic landmarks. Previously defined hexamers enriched around TDP-43 iCLIP binding sites^6^ (Supplementary Fig. 6a) were overrepresented among the most enriched hexamers proximal to all cryptic landmarks, with the strongest signal overall observed at both the 3’ splice site (3’ss) and PAS of ALE events (Supplementary Fig. 6b). To assess the concordance with iCLIP binding profiles, we visualized the positional coverage of the hexamer group most strongly associated with TDP-43 binding^6^. For ALEs, we observed a notable peak immediately upstream of splice acceptors and a strong peak downstream of PAS (Fig. 1d), although previous reports of splice-site-dependent STMN2 cryptic ALE repression^33^ suggest that the binding at PAS may have secondary effects. Enriched signal was also observed immediately downstream of the distal PAS of 3’Exts (Fig. 1e).

**Extended Data Fig. 2:**
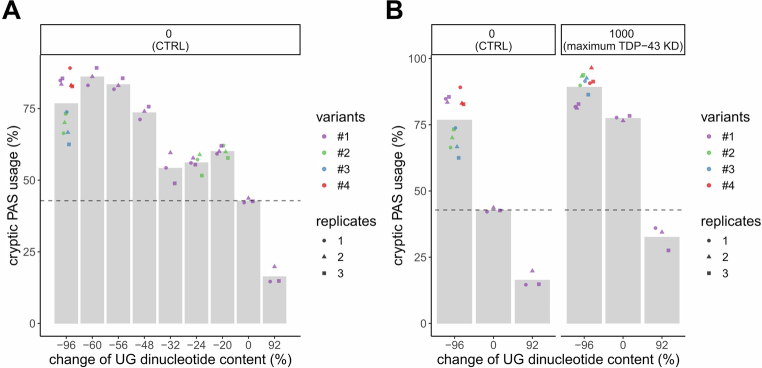
ELK1 3’-UTR APA reporter library. **A**). ELK1 Cryptic PAS usage in control (‘CTRL’) conditions in a series of reporters with varying changes in UG content (x-axis, %). Original reporter (0); reporters with increasing amounts of UG deletion (20, 24, 32, 48, 56, 60, 96); the reporter where UG content is increased (92). **B**). Cryptic PAS usage in control and severe TDP-43 knockdown conditions for the original reporter (0), the reporter with increased UG content (92) and the reporter with the most UG deletion (96).

To experimentally validate the direct relationship between TDP-43 binding and cryptic PAS usage, we generated a reporter for the *ELK1* 3’Ext APA (Fig. 1f). Nanopore sequencing showed a strong upregulation of the distal cryptic PAS upon TDP-43 knockdown in neuronal cells (Fig. 1g), confirming similar behavior to endogenous *ELK1*. We then focused on 150 base pairs downstream of the constitutive poly(A) site, where iCLIP data show TDP-43 binding to occur, and generated a series of constructs where we removed or increased UG content to disrupt or enhance TDP-43 binding (Fig. 1f). Under normal TDP-43 levels, cryptic PAS usage was enhanced by UG depletion, whereas it was reduced by UG dinucleotide content increase (Extended Data Fig. 2a,b). Increasing levels of TDP-43 knockdown enhanced cryptic PAS usage in constructs with normal, increased or moderately disrupted UGs, whereas constructs with severe UG depletion did not respond to TDP-43 depletion, confirming a direct regulation by TDP-43 (Fig. 1h).

Overall, our data support a direct role for TDP-43 binding in both enhancing and repressing PAS usage, therefore leading to cryptic APA upon TDP-43 loss.

### TDP-43 cryptic APA is detectable in postmortem ALS/FTD tissues

**Fig. 2:**
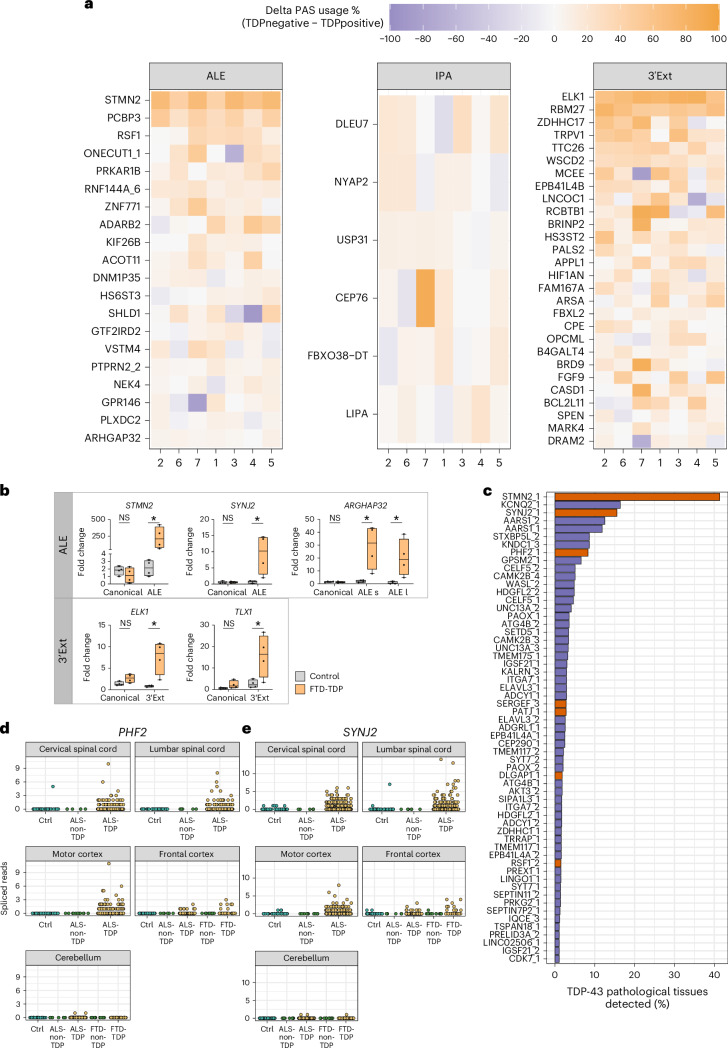
Cryptic APAs are detected in postmortem ALS/FTD RNA-seq datasets. **a**, Heatmap of cryptic last exon usage in postmortem FACS-seq data^34^. Cells are colored according to the magnitude of sample-wise difference in usage between TDP-43-depleted (TDPnegative) and TDP-43-positive (TDPpositive) cells. Rows represent individual cryptic last exons from in vitro that passed enrichment criteria (median sample-wise difference in usage (TDPnegative − TDPpositive) > 5%) and are arranged in descending order of the difference in usage within each event type. Columns represent individual patients within the cohort. **b**, RT−qPCR analysis after 3’RACE for the indicated 3’UTRs in frontal cortex samples of control patients (*n* = 4) and FTD (FTD-TDP, *n* = 4) cases with TDP-43 pathology. The RNA expression levels were normalized against *GAPDH* mRNA and expressed as relative fold change with respect to one control sample set to a value of 1. *PHF2* and *SIX3* genes (shown in Supplementary Fig. 3) were excluded owing to unspecific amplification of the cryptic isoforms in tissues. Data are represented as box plots (lower, middle and upper quartiles), and error bars span from the minimum to the maximum value. Two-sided Studentʼs unpaired *t*-test (NS *P* > 0.05, *P < 0.05). l, long; s, short. *STMN2* *P* = 0.330 (canonical), 0.033 (ALE). *SYNJ2* *P* = 0.847 (canonical), 0.031 (ALE). *ARHGAP32* *P* = 0.500 (canonical), 0.021 (ALE s), 0.035 (ALE l). *ELK1* *P* = 0.056 (canonical), 0.013 (3’Ext). *TLX1* *P* = 0.130 (canonical), 0.041 (3’Ext). All *P* values are to 3 decimal places (d.p.). **c**, Selectively expressed cryptic ALEs (orange) and splicing events^13^ (purple) in tissues and samples with TDP-43 proteinopathy in the NYGC ALS Consortium dataset. Events are considered detected if at least two junction reads were detected in a sample. **d**, Detection of spliced reads for the cryptic ALE in *PHF2* across samples in the NYGC ALS Consortium dataset. Color indicates whether disease subtype and region is expected (orange) or not expected (green) to have TDP-43 pathology and cryptic spliced read expression. **e**, As in **d** but for cryptic ALE in *SYNJ2*. NS, not significant. Source data

We next investigated whether the cryptic APA detected in vitro occurred also in postmortem central nervous system (CNS) tissue samples affected by TDP-43 proteinopathy. We initially focused on neuronal nuclei sorted into TDP-43-positive and TDP-43-negative populations^34^. Fifty-four cryptic APA events were more highly expressed in TDP-43-depleted nuclei. All APA event types were represented in this list (MEP_L_fig2; Fig. 2a), with ALEs (20) and 3’Exts (28) representing the majority of enriched events. Our analysis confirmed previously reported cryptic ALEs with patient specificity, such as in *STMN2* (ref. ^35^). Numerous 3’Exts also show enrichment in TDP-43-negative nuclei in a similar magnitude to *STMN2* (median increased usage of 69%), most notably *ELK1* (76%) and *RBM27* (57%) (Fig. 2a). Six IPA events meet our enrichment criteria (Fig. 2a), including *USP31*, which was identified in a targeted assay of sporadic ALS motor cortex tissue^36^. However, IPA events were generally more weakly enriched in TDP-43-depleted nuclei compared to 3’Ext and ALE events. We validated the occurrence of cryptic APAs by performing 3’RACE in FTD frontal cortex samples (Fig. 2b and Supplementary Fig. 7). Altogether, this analysis shows that cryptic APA is detectable in postmortem ALS/FTD CNS.

**Extended Data Fig. 3:**
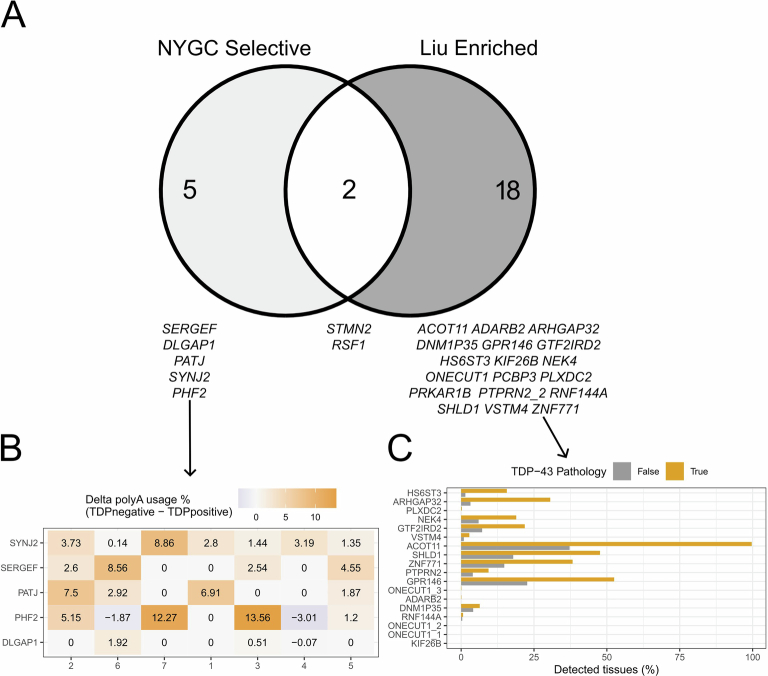
Consistency of enriched/selective ALEs between FACS-seq and NYGC datasets. **A**). Overlap between ALEs passing enrichment threshold in the ‘Liu’ FACS-seq data^34^ (Fig. 2a) and splice junctions of ALEs passing selective detection thresholds in the New York Genome Centre (NYGC) ALS Consortium dataset (Fig. 2b). Cryptic ALEs in each intersection group are labelled directly underneath the event count. **B**). Heatmap of PAS usage in post-mortem FACS-seq data^34^ for NYGC-specific ALEs. Cells are labelled with and coloured in proportion to the magnitude of the sample-wise difference in PAS usage between TDP-43 depleted (TDPnegative) and TDP-43 positive (TDPpositive) nuclei. Rows are arranged in descending order of the median sample-wise difference in usage (TDPnegative - TDPpositive). Columns represent individual patients within the cohort. **C**). Detection statistics for FACS-seq specific ALEs in the NYGC ALS Consortium. ALEs are sorted in descending order of the detection enrichment ratio and bars are coloured according to expected presence (gold, ‘True’) or absence (grey, ‘False’) of TDP-43 proteinopathy. ALEs are considered detected if at least 2 junction reads were present in a sample.

Next, we used the New York Genome Center (NYGC) ALS Consortium RNA-seq dataset to assess cryptic APA in a larger cohort of CNS cases with or without TDP-43 pathology (Supplementary Table 4). Cryptic 3’Exts often demonstrated low basal expression in control samples in our in vitro datasets, confounding the detection in postmortem bulk RNA-seq datasets, in which only a very small proportion of cells is expected to have TDP-43 pathology. IPA detection is further complicated by the fact that normal pre-mRNA reads also map to IPA regions, creating significant noise in bulk RNA-seq. We, therefore, focused on ALEs, where detection of the associated upstream cryptic splice junctions provide direct evidence of expression. As cryptic ALEs are expected to be dependent on nuclear TDP-43 depletion, we defined criteria based on spliced read detection to identify cryptic events with specific expression in tissues and disease subtypes where TDP-43 pathology is present. Of 118 cryptic ALE junctions, 7 fulfilled specificity criteria (Supplementary Table 5), in contrast to 56 out of 313 cryptic splicing events collated from i3Neurons with TDP-43 knockdown^13^ (Fig. 2c and Extended Data Fig. 3). *STMN2* was most frequently detected in tissues with expected TDP-43 proteinopathy, and several other ALEs were among the most frequently detected specific cryptic events, including *SYNJ2* (third; Fig. 2d) and *PHF2* (eighth; Fig. 2e).

Altogether, this suggests that cryptic APAs are detectable in postmortem tissue affected by TDP-43 pathology, highlighting their potential relevance in loss-of-function disease mechanisms and their promising utility as biomarkers.

### Cryptic APA events variably affect differential expression

Cryptic splicing events impact expression, often leading to a reduction in transcript levels^9–11^. We, therefore, assessed the effect of cryptic APAs on their own transcripts in i3Neurons^13^ (Supplementary Fig. 8a) and found that the majority of events (86 out of 126) coincide with a significant change in expression, equally split between significant upregulation and downregulation. When subdivided further into cryptic APA categories, no category showed a clear bias for upregulation or downregulation (19 out of 34 3’Ext, 17 out of 37 ALE and 6 out of 10 IPA genes are downregulated). This suggests that cryptic APAs are associated with differential expression but have variable effects on transcript levels.

### Cryptic 3’Ext events can lead to increased translation and function

Regulation of both ALE and 3’Ext usage has been demonstrated to impact protein abundance through distinct mechanisms^37,38^, but differential RNA abundance does not necessarily imply a coordinated change in protein levels. To assess whether changes in gene expression were also reflected in translation levels, we performed differential translation analysis of Ribo-seq data generated from i3Neurons with TDP-43 depletion^13^.

**Fig. 3:**
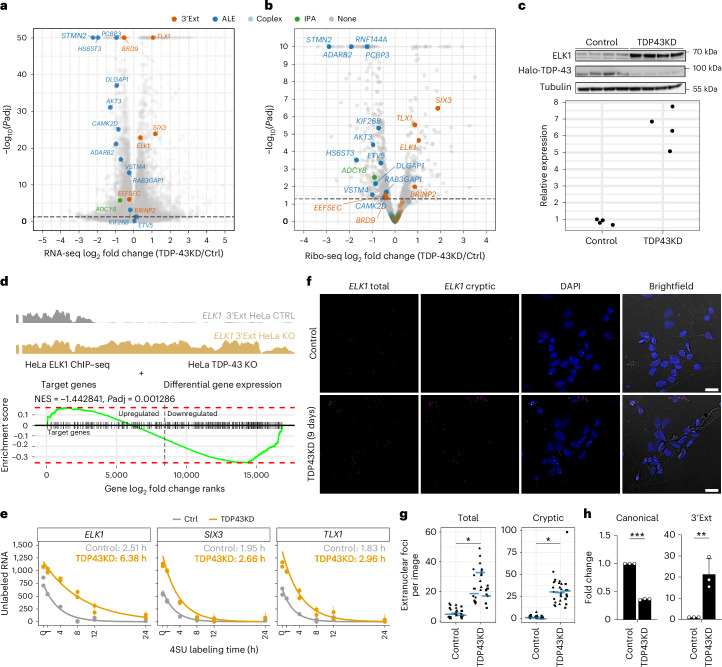
Cryptic 3’ UTR extensions in transcription factor RNAs lead to increased RNA and protein levels by increased RNA stability and cytoplasmic RNA levels. **a**, RNA-seq differential expression volcano plot (TDP-43 knockdown versus control i3Neurons). Cryptic 3’Ext (orange), ALE (blue) and IPA (green) containing genes with increased translation (Fig. 3b) are colored and labeled. *y* axis 50, −log_10_-adjusted *P* (*P*adj) ≥ 50. **b**, Ribo-seq differential expression volcano plot (TDP43KD versus CTRL i3Neurons). Colors: cryptic 3’Ext (orange), ALE (blue) or IPA (green) containing genes. *y* axis 10, −log_10_-adjusted *P* ≥ 10. **c**, ELK1 protein levels in Halo-TDP-43 i3Neurons^61^. Top, ELK1 western blot showing increased ELK1 protein expression upon TDP-43 knockdown (*n* = 4 independent differentiations). Bottom, tubulin-normalized ELK1 band intensities (**c**, top) in control and TDP43KD Halo-TDP-43 i3Neurons. **d**, ELK1 transcription factor activity. Top, *ELK1* cryptic 3’Ext RNA-seq coverage traces in control (black) and TDP-43 knockout (KO) (gold) HeLa cells^49^. Bottom, GSEA enrichment plot for ChIP–seq-defined ELK1 target genes in TDP-43 knockout HeLa cells. Green line denotes GSEA enrichment statistic; red lines denote maximum value in upregulated (left) and downregulated (right) genes; black lines denote ELK1 target genes (*n* = 353). NES is relative to mean score of identically sized, randomly sampled gene sets. **e**, Decay curve for RNA produced before 4SU labeling (old) in control (gray, 4 h *n* = 1, others *n* = 2) and knockdown (orange, all *n* = 2) i3Neurons. Curves denote fitted estimate of old RNA levels. Points denote old RNA abundance estimates. Error bars denote upper and lower 95% credible interval. Inset text shows the gene-level GrandR-estimated half-lives. **f**, Representative images for FISH probes targeting the annotated (*ELK1* total, green) 3’UTR and cryptic 3’UTR-specific (*ELK1* cryptic, magenta) *ELK1* sequences in control (top row) and TDP-43 knockdown (bottom row) i3Neurons. Scale bars, 10 µm. **g**, Extranuclear FISH signals for the *ELK1* total and cryptic probes. Points denote foci counts (*n* = 10 images). Blue bars denote mean count. Two-sided, one-sample *t*-test after within-replicate control normalization (*n* = 3, *P < 0.05, total *P* = 0.009, cryptic *P* = 0.012 (3 d.p)). **h**, *ELK1* canonical and cryptic (3’Ext) isoform 3’RACE and RT−qPCR of the cytoplasmic fraction of TDP-43-depleted SH-SY5Y cells. Bars denote mean fold change versus control cells ± s.d. (*n* = 3 biological replicates). Two-sided Studentʼs unpaired *t*-test (**P = 0.009, ***P = 7.535 × 10^−8^). Source data

Only a minority of cryptic APA-containing genes (26 out of 126) showed significant changes in overall translation levels (Supplementary Table 6), of which 24 are concordantly altered in both Ribo-seq and RNA-seq abundance upon TDP-43 knockdown, including previously reported STMN2 (refs. ^39,40^) (Fig. 3a,b). Notably, the differentially translated subset appeared to stratify by APA category: whereas ALEs are downregulated, all four significant 3’Exts, which also showed increased RNA abundance (Fig. 3a), had significantly increased translation (Fig. 3b). Gene set enrichment analysis (GSEA)^41,42^ confirmed that cryptic ALE and 3’Ext genes are significantly associated with decreased translation (normalized enrichment score (NES) −2.09, adjusted *P* = 2.31 × 10^−6^) and increased translation (NES 1.54, adjusted *P* = 0.03), respectively, whereas IPA genes show no significant association in either direction (NES −1.09, adjusted *P* = 0.36) (Supplementary Fig. 8b).

Interestingly, the three 3’Ext-containing genes that were most upregulated at both RNA and translation levels (Fig. 3a,b) encode for three transcription factors: *ELK1*, *SIX3* and *TLX1*. The regulation of these 3’Ext events is reproducible across in vitro datasets (Supplementary Fig. 1). As *ELK1* increase was previously associated with neuronal toxicity^43–45^ and its levels are consistently higher in mature neurons, compared to *SIX3* and *TLX1*, which are associated with neuronal development^46,47^, we decided to focus our investigations on *ELK1*. We tested whether the increase in Ribo-seq also corresponded to an upregulation of steady-state protein, and western blots confirmed a significant increase in ELK1 protein expression upon TDP-43 knockdown in i3Neurons (Fig. 3c). We next asked whether the activity of ELK1, which functions as a transcription factor in the ternary complex factor (TCF) family^48^, could be altered in the context of TDP-43 loss. We assessed whether ELK1 target genes defined by chromatin immunoprecipitation followed by high-throughput sequencing (ChIP–seq) in HeLa cells were also affected in TDP-43 knockout HeLa cells^49^, in which the cryptic 3’Ext is robustly upregulated (Fig. 3d). Using GSEA, we observed a significant change in ELK1 target gene expression upon TDP-43 knockout (Fig. 3d). This suggests that cryptic 3’Exts can lead to change in function in the context of TDP-43 loss.

### Transcription factors with cryptic 3’Ext events have increased RNA stability

We investigated the mechanisms by which cryptic 3’UTRs could mediate increased translation levels of *ELK1*, *SIX3* and *TLX1*. We revisited differential splicing analysis of i3Neuron RNA-seq datasets^10,13^ and confirmed that cryptic 3’Exts are the only differential RNA processing events occurring in these three transcription factor RNAs upon TDP-43 depletion. As alternative 3’UTRs have been linked to differences in RNA stability^50^, we reasoned that increased RNA stability could account for changes in overall RNA abundance and translation levels. To investigate changes in RNA stability in i3Neurons with TDP-43 depletion, we performed SLAM-seq^51^, which allows the detection of newly synthesized RNAs through incorporation of a uridine analogue (4SU). Different lengths of 4SU treatment allow the estimation of gene-level RNA half-lives. We observed increased half-lives in cryptic 3’Ext-containing genes *ELK1*, *TLX1* and *SIX3* (Fig. 3e). To confirm that the 3’Ext half-life change was due to the cryptic APA event, we performed an isoform-specific analysis for *ELK1* in control i3Neurons, where the distal (cryptic) 3’Ext is sufficiently expressed to be analyzed and not prevalent enough to confound the evaluation of the proximal (shared) isoform. We observed elevated *ELK1* 3’Ext half-life relative to the proximal PAS (Supplementary Fig. 9a). Altogether, this suggests that increased RNA abundance and translation of cryptic 3’Ext genes are mediated by increased RNA stability.

**Extended Data Fig. 4:**
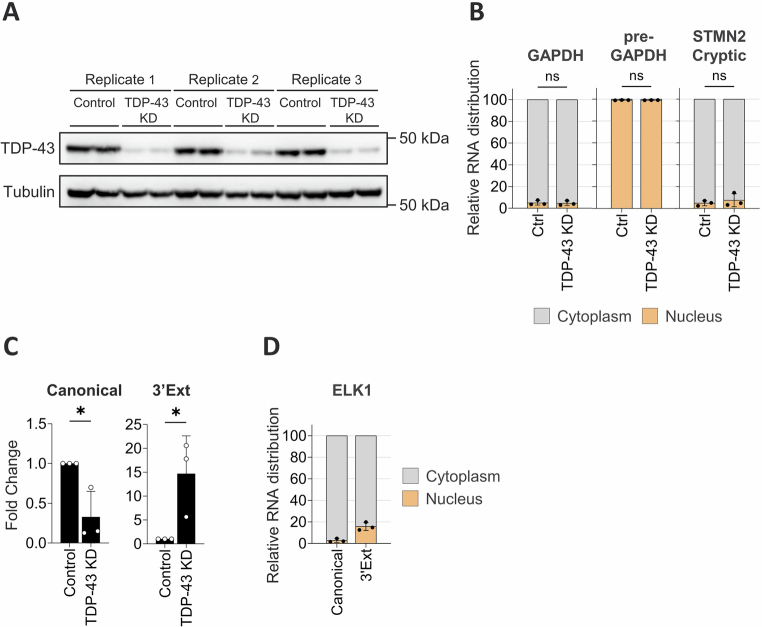
Subcellular fractionation of SH-SY5Y upon TDP-43 depletion. **A**). Western blots to evaluate the decrease of TDP-43 protein upon its depletion in SH-SY5Y cell line; Tubulin was used as loading control. For each experimental condition, two technical replicates were loaded on the gel. n=3 biological replicates. **B**). Bar-plots showing the percentage in the nuclear and cytoplasmic fractions in SH-SY5Y cell line for selected targets in control condition (“Ctrl”) or upon TDP-43 depletion (“TDP-43 KD”) detected through qRT-PCR analysis. GAPDH and pre-GAPDH were used as cytoplasmic and nuclear controls, respectively, for cell fractionation. STMN2 Cryptic, a well-reported cryptic exon, shows predominant cytoplasmic localization. The relative RNA distribution in the bars is represented as mean ± standard deviation. n=3 biological replicates. Statistical analyses were performed using Student unpaired t-test (n.s. p>0.05, * p<0.05, ** p<0.01, **** p<0.0001). GAPDH p-value (3 d.p.): 0.865, pre-GAPDH p: 0.936, STMN2 Cryptic p: 0.516. **C**). RT-qPCR analysis after 3’RACE on the nuclear fraction of SH-SY5Y cell line upon TDP-43 depletion (“TDP-43 KD”). The levels of ELK1 canonical (“Canonical”) and cryptic (“3’Ext”) isoforms are expressed as relative fold change with respect to the control condition (“Control”) set to a value of 1. Data are represented as the mean of the fold change ± standard deviation. n=3 biological replicates. Statistical analyses were performed using Student unpaired t-test (* p<0.05). 3’Ext: 3’UTR extension. ELK1 Canonical p-value (3 d.p.): 0.023, ELK1 3’Ext p: 0.041. **D**). Bar-plots showing the percentage in the nuclear and cytoplasmic fractions in SH-SY5Y cell line upon TDP-43 depletion for ELK1 canonical (“Canonical”) and cryptic (“3’Ext”) isoforms, as detected through qRT-PCR analysis. The relative RNA distribution in the bars is represented as mean ± standard deviation. n=3 biological replicates. 3’Ext: 3’UTR extension. Source data

Given that translation depends on extranuclear localization of mRNAs, we tested whether cryptic 3’Ext transcripts localize to the cytoplasm and contribute to the increased translation levels^52–55^. Focusing on the *ELK1* cryptic 3’Ext, we designed probes to recognize the common proximal sequence and the distal sequence specific to the 3’Ext and performed fluorescence in situ hybridization (FISH) in i3Neurons where we could detect both probes in the nuclei, cytoplasm and neurites (Fig. 3f). Consistent with RNA-seq, we observed a significant increase in total foci for both the total and cryptic-specific probes upon TDP-43 knockdown (Fig. 3g and Supplementary Fig. 9b,c). To specifically discriminate and quantify proximal and distal *ELK1* APA subcellular localization, we performed 3’RACE on SH-SY5Y cells after nuclear cytoplasmic fractionation. We found that both isoforms are predominantly localized to the cytoplasm and that, upon TDP-43 knockdown, the proximal canonical APA is reduced, whereas the cryptic 3’Ext is increased (Fig. 3h and Extended Data Fig. 4). Finally, we evaluated ELK1 isoform-specific ribosome recruitment using fractionation and sequencing (Frac-seq) data from neural progenitor cells^56^. We found *ELK1* cryptic 3’Ext to be relatively enriched in ribosome-associated fractions, supporting a preferential engagement of the cryptic 3’Ext with the translation machinery (Supplementary Fig. 9d). Overall, these findings show that ELK1 cryptic 3’Ext has increased RNA stability, localizes to the cytoplasm and neurites and is translated, driving the increase in ELK1 protein.

## Discussion

Defining TDP-43 RNA targets is critical to understanding the molecular consequences of nuclear TDP-43 depletion. Thus far, efforts have mainly focused on the consequences of altered splicing and have successfully identified key targets that are being pursued as therapeutic targets and potential biomarkers for TDP-43 pathology^10,11,14,39,40^. Although TDP-43 is involved in multiple aspects of RNA processing, including polyadenylation^4–6^, this has been largely understudied due to the lack of effective tools to address these questions. Furthermore, although splicing analyses were able to identify ALE events (for example, *STMN2*) because of the upstream novel splice junction, they would not detect novel IPA and 3’Ext events. Here, we developed a pipeline to detect and quantify novel APA events from total RNA-seq and apply it to a wide range of neuronal TDP-43 loss-of-function datasets to define cryptic APAs, a novel category of cryptic RNA processing events of potential relevance to ALS/FTD. iCLIP and TDP-43 binding motif analyses support a direct regulation of these events by TDP-43, in which TDP-43 loss can both weaken conventional poly(A) sites and de-repress cryptic APA. Similar to splicing, where TDP-43 can both repress or enhance exon inclusion, TDP-43 can, therefore, have a dual action on transcript termination. Notably, for disease relevance, and similar to cryptic splicing, numerous cryptic APA events can be detected in postmortem tissue and are specifically expressed upon TDP-43 pathology.

We then moved to investigate the impact of cryptic APAs on RNA levels and translation and found that IPAs and ALEs either had no impact or induced a reduction of transcript levels in RNA-seq and Ribo-seq analyses—in line with previous observations on known cryptic ALEs such as *STMN2* (refs. ^39,40^). Recent work demonstrated that cryptic exon-containing transcripts can be translated and produce cryptic peptides that could serve as biomarkers of TDP-43 pathology^13,14^. As cryptic ALE and IPA events are mostly predicted to be insensitive to nonsense-mediated decay, and are located often within the coding sequence, they are likely to give rise to cryptic peptides; for example, cryptic ALE *RSF1* encodes a cryptic peptide that is detected in the cerebrospinal fluid of patients with ALS^13^. Previous work identified cryptic ALEs, as their novel splice junction can be detected by numerous splice detection packages^13,35,57^. Conversely, IPAs have been more difficult to identify, and further work should consider whether these cryptic IPA events can be detected in patient brains and biofluids as an indirect measure of TDP-43 pathology.

Surprisingly, 3’Ext events in the three transcription factor-encoding genes *ELK1*, *SIX3* and *TLX1* were associated with transcript upregulation and increased translation and protein levels. We found this to be associated with an increase in RNA stability. Thus, in contrast to the conventional model of TDP-43-regulated cryptic splicing leading to reduced protein levels or to altered proteins containing cryptic peptides, cryptic 3’Ext can be associated with increased protein levels, outlining a novel consequence of TDP-43 cryptic RNA processing.

*ELK1*, *SIX3* and *TLX1* 3’Ext are reliably induced upon TDP-43 depletion across our in vitro datasets, suggesting that they are not cell-type-specific, sensitive TDP-43 targets. These three transcription factors have been studied in the neuronal context, although *SIX3* and *TLX1* are primarily expressed in the developmental stage^46,47^. Our work, therefore, focused on *ELK1*, and we were able to validate the cryptic 3’Ext in patient brains both by 3’RACE and by analysis of publicly available data, whereas detection of increased protein levels is more challenging due to ELK1 being expressed ubiquitously and TDP-43 pathology occurring in only in a minority of cells. We were also able to use HeLa cell data to show that TDP-43 loss can induce changes in *ELK1* target genes. *ELK1* promotes axonal outgrowth^58^ and is increased in Huntington’s disease models where it can have a neuroprotective role^59^. *ELK1* overexpression has also been linked with neurotoxicity through interaction with components of the mitochondrial permeability−transition pore complex^44^, and dendrite-specific overexpression of *ELK1* mRNA induced cell death in a transcription-dependent and translation-dependent manner^43^, supporting a potential contribution of this cryptic APA to pathogenesis. Further work is needed to investigate the functional relevance of increased *ELK1*, *SIX3* and *TLX1* expression in models of TDP-43 proteinopathy.

We focused on identifying cryptic APA events, as their extreme expression changes upon TDP-43 loss render them favorable therapeutic and biomarker targets. As reported in the accompanying manuscript by Zeng et al.^60^, the authors investigated APA dysregulation more generally upon TDP-43 loss and show that it is widespread (in accordance with our findings in Fig. 1b), can occur in ALS/FTD-related genes^60^ and can lead to change in function^29^, underscoring the potential relevance of APA in disease pathogenesis. We note that several targets (for example, *CNPY3*, *ELK1* and *ARHGAP32*) are commonly identified across the studies despite diverging methodological approaches, underlying the consistency of our observations. Notably, similar to our findings for *ELK1*, *SIX3* and *TLX1*, both Zeng et al.^60^ and Arnold et al.^29^ also found that APAs can lead to upregulation of normal protein levels, consolidating this as a general consequence of TDP-43 loss. Our studies collectively demonstrate that dysregulated APA is a general consequence of nuclear TDP-43 loss in ALS/FTD. Beyond mRNA and protein levels, APA can impact RNA localization and local translation, and targeted work will be necessary to comprehensively identify and detect these alterations.

In summary, we provide a compendium of cryptic APA events determined by TDP-43 loss as a resource for studying RNA dysregulation and identifying novel biomarkers in ALS. Our work also shows that cryptic RNA processing can lead to an increase in protein expression and function, expanding the molecular consequences of TDP-43 loss and pathology, with implications for disease pathogenesis and therapeutic target identification.

## Methods

A summary table mapping cellular models to their respective analyses is provided in Supplementary Table 9.

### CRISPR interference knockdown in human iPSCs and differentiation and culture of i3Neurons

CRISPR interference (CRISPRi) knockdown experiments were performed in the WTC11 iPSC line harboring stable TO-NGN2 and in dCas9−BFP−KRAB cassettes at safe harbor loci^62^. CRISPRi knockdown of TDP-43 in iPSCs was achieved using single guide RNA (sgRNA) targeting the transcription start site of TARDBP (or non-targeting control sgRNA)^10^, delivered by lentiviral transduction. sgRNA sequences were as follows: non-targeting control GTCCACCCTTATCTAGGCTA and TARDBP GGGAAGTCAGCCGTGAGACC. iPSCs were differentiated into cortical-like i3Neurons as described previously^10,63^ and fixed 9 days after re-plating for RNA-FISH.

For RNA-seq experiments (‘Humphrey i3 cortical’), i3Neurons were induced as previously described^63^ with the addition of SMAD and WNT inhibitors^64^ (SB431542 10 µM; LDN-193189 100 nM; XAV939 2 µM, all from Cambridge Bioscience). After induction, cells were cultured in BrainPhys Media (STEMCELL Technologies) with 20 ng ml^−1^ BDNF (PeproTech), 20 ng ml^−1^ GDNF (PeproTech), 1× N2 supplement (Thermo Fisher Scientific), 1× B27 supplement (Thermo Fisher Scientific), 200 nM ascorbic acid (Sigma-Aldrich), 1 mM dibutyryl cyclic-AMP (Sigma-Aldrich) and 1 µg ml^−1^ laminin (Thermo Fisher Scientific), as previously described^65^, and harvested 30 days after differentiation. The ‘Zanovello i3 Cortical’ samples were generated as previously described for the dual TDP-43/UPF1 knockdown experiments^10^. Only the TDP-43/Control and Control/Control transfection conditions were used for RNA-seq. See the ‘RNA-seq’ section for library preparation details.

An iPSC line with an N-terminal HaloTag on both endogenous copies of TDP-43 (Halo-TDP-43 i3Neurons) was generated by CRISPR−Cas12 gene editing^61^. The parental cell line used was the WTC11 cell line with integrated dCas9−Krab and NGN2 cassettes as mentioned previously^62^. The homology-directed repair (HDR) template used was Addgene plasmid 178131. Editing was done with Cas12 CRISPR RNA (crRNA) (Integrated DNA Technologies) with GGAAAAGTAAAAGATGTCTGAAT as the targeting sequence. Recombinant Cas12 (Cpf1 ultra; Integrated DNA Technologies) was electroporated with HDR template and Cas12 crRNA using the P3 Primary Cell 4-D Nucleofector Kit (Amaxa, V4XP-3024). iPSCs were then single-cell plated, and positive colonies were selected with HaloTag TMR dye (Promega) and verified by polymerase chain reaction (PCR) of genomic DNA.

For proteolysis-targeting chimera (PROTAC)-mediated knockdown of Halo-TDP-43, i3Neurons were treated with HaloPROTAC-E^66^ (30 nM) on days in vitro 14 (DIV14) and harvested on DIV28. This protocol allows to avoid incurring in maturation alterations caused by loss of TDP-43, as this occurs at a later step; we, therefore, used this approach to validate ELK1 protein increase as transcription factor levels can be sensitive to maturation stages.

### FISH

Cortical-like i3Neurons were cultured on 13-mm glass coverslips and fixed in 4% paraformaldehyde (PFA)/sucrose on day 9. RNA-FISH was performed using the QuantiGene ViewRNA ISH Cell Assay Kit (Invitrogen, QVC0001), according to the manufacturer’s instructions. Protease was used at 1:1,000 dilution. Two probe sets were used to detect the canonical *ELK1* transcript (TYPE 4 probe, 488-nm) or specifically the distal 3’UTR cryptic extension (TYPE 1 probe, 550-nm). Confocal images were acquired with an LSM 980 laser scanning confocal microscope with Airyscan 2 (Zeiss), using a ×40 oil immersion objective.

For each biological replicate, 10 images were acquired for the control and TDP-43 knockdown conditions. For each image, foci for both probes were counted within the 106.07-µm × 106.07-µm field of view on FIJI/ImageJ using the maximum intensity *z*-projection function to flatten the 2-µm-thick *z* stack. The ‘Find Maxima’ function using the same prominence setting between conditions was performed to quantify total numbers of RNA foci. To separately count nuclear and cytoplasmic foci, the Cell Counter plugin was used. For each probe and field of view, the total number of foci was divided by the number of DAPI-stained nuclei to give the average number of foci per cell. To calculate the nuclear:extranuclear ratio for the ‘Total *ELK1*’ probe, the number of nuclear foci was divided by the number of extranuclear foci in each field of view. For each probe and condition, the mean number of foci per cell and the nuclear:extranuclear ratio were calculated from the 10 images and normalized, for each biological replicate, to the respective control condition. Statistical significance was evaluated using a one sample *t*-test with a log transformation and the Benjamini− Hochberg false discovery rate procedure, testing the null hypothesis that mean = log(1).

### Western blots

Halo-TDP-43 i3Neurons were homogenized in lysis buffer (25 mM Tris-HCl, 150 mM NaCl, 1% NP-40, 1% glycerol, 2 mM EDTA, 0.1% SDS, protease inhibitor (cOmplete EDTA-free protease inhibitor cocktail; Roche) and phosphatase inhibitor (PhoSTOP; Roche)). Samples were loaded on a NuPAGE 4−12% Bis-Tris protein gel (Invitrogen), which was run in NuPAGE MOPS buffer. Proteins were transferred onto PVDF blotting membrane (Amersham) through wet transfer for 1 hour and 30 minutes at 200 mA in transfer buffer (25 mM Tris, 192 mM glycine and 20% methanol). The membrane was blocked in 5% milk in TBST (20 mM Tris, 150 mM NaCl and 0.1% Tween 20) and incubated overnight with primary antibodies diluted in 5% milk in TBST (anti-ELK1 (Abcam, ab32106) 1:500, anti-TDP-43 (Abcam, ab104223) 1:2,000 and anti-tubulin (Sigma-Aldrich, MAB1637) 1:5,000). After 1-hour incubation with horseradish peroxidase (HRP)-conjugated secondary antibodies diluted in 5% milk in TBST (anti-mouse HRP (Bio-Rad, 1706516) 1:10,000 and anti-rabbit HRP (Bio-Rad, 1706515) 1:10,000), the membrane was developed using Immobilon Classico HRP substrate (Sigma-Aldrich) and the Bio-Rad ChemiDoc system.

### Cell fractionation

For the fractionation experiments, SH-SY5Y cells were treated for 10 days with 25 ng ml^−1^ doxycycline hyclate (Sigma-Aldrich) to induce the short hairpin RNA (shRNA) against TDP-43. After 10 days, cells were trypsinized, pelleted and resuspended in 1× PBS. Before re-pelletting them, a fraction for each sample was saved for protein analysis to assess TDP-43 depletion. The other fraction was used for the subcellular fractionation with the Ambion PARIS Kit (Life Technologies), according to the manufacturer’s instructions. RNA from the nuclear and cytosolic fractions was extracted with the Direct-zol kit (Zymo Research) with on-column DNase I treatment. For each experimental condition, 2 μg of cytoplasmic RNA and an equal volume of nuclear RNA fraction were reverse transcribed with the RevertAid First Strand cDNA Synthesis Kit (Thermo Fisher Scientific) according to the manufacturer’s instructions and analyzed by RT−qPCR with PowerUp SYBR Green Master Mix (Thermo Fisher Scientific). DNA amplification was monitored on a QuantStudio 5 Real-Time PCR system (Applied Biosystems). GAPDH and pre-GAPDH transcripts were used as cytosolic and nuclear controls, respectively. The oligonucleotides used for the analyses are reported in Supplementary Table 9.

### 3’RACE

For each condition, equal amounts of total RNA were reverse transcribed in a 20-µl reaction with the RevertAid First Strand cDNA Synthesis Kit (Thermo Fisher Scientific), according to the manufacturer’s instructions, using 1 µl of 50 µM oligo dT-anchor RT primer. cDNAs were diluted to 1 ng µl^−1^, and the expression of each target was evaluated through RT−qPCR with PowerUp SYBR Green Master Mix (Thermo Fisher Scientific) using a gene-specific forward primer and the PCR universal reverse primer. DNA amplification was monitored on the QuantStudio 5 Real-Time PCR system (Applied Biosystems). Unless otherwise specified in the figure legend, relative RNA quantity was calculated as the fold change (2^−ΔΔCt^) with respect to the experimental control sample set as 1 and normalized over GAPDH, used as an endogenous control. The oligonucleotides used for the analyses are reported in Supplementary Table 8.

### ELK1 3′UTR APA reporter library

For the initial test, we cloned the ELK1 proximal 3’UTR and the first 800 bp of ELK1 cryptic 3’Ext into the region downstream of the mGreenLantern coding sequence in a dual-fluorescent (mScarlet and mGreenLantern), dual-promoter reporter plasmid. We then transfected two groups of SK-N-BE(2) cells with our construct: one treated with 1,000 ng ml^−1^ doxycycline and one untreated, and each group had triplicates. This cell line contains the SMARTvector, which enables TDP-43 knockdown upon doxycycline treatment. One day after transfection, we combined triplicates together for RNA extraction and performed 3’RACE to generate DNA samples. Subsequently, we submitted these samples for nanopore sequencing and analyzed the sequencing data to assess APA site usage. First, we used minimap2 version 2.28 (ref. ^67^) to perform alignment. Subsequently, we determined the polyadenylation site for each read by locating the sequence of 10 consecutive adenosines and their corresponding position in the alignment reference.

For the subsequent UG replacement experiment, we constructed a plasmid library. This cloning included three steps. (1) We inserted a restriction site between the mGreenLatern coding sequence and ELK1 3’UTR within the construct. (2) We digested the construct with AfeI (New England Biolabs, R0652) and AccI (New England Biolabs, R0161), whose cutting sites are located at proximal 3’UTR and cryptic 3’Ext, respectively. Next, using Gibson assembly^68^, we assembled the digested plasmid backbone with the inserts (described below) to produce the library. Plasmids with different inserts were referred to as variants. (3) We used the restriction site inserted in the first step to incorporate a 15-mer random barcode into each variant. After this, each variant in the library corresponded to one or more unique barcodes, which could be used to identify inserts during sequencing data analysis.

Each insert consisted of three distinct fragments: the first fragment comprised the last 192 bp of ELK1 proximal 3’UTR, whereas the second and third fragments comprised the first 350 bp of ELK1 cryptic 3’Ext. Moreover, the first and last 28 bp of each fragment were conserved to enable Gibson assembly with adjacent fragments and the plasmid backbone. To emphasise the importance of the first 150 bp of cryptic 3’Ext within the second fragment, we focused on it in our results.

Before transfection, we conducted nanopore sequencing to identify each variant’s corresponding unique barcodes. We followed the protocol described above to transfect the plasmid library into SK-N-BE(2) cells with SMARTvector in four treatment groups (0, 30 ng ml^−1^, 60 ng ml^−1^ and 1,000 ng ml^−1^ doxycycline). The protocol was performed in triplicate for each variant, and replicates were not combined before RNA extraction. After obtaining the nanopore sequencing results, we used a custom script to extract the barcode sequence from each read to identify which insert the read should be aligned to. Reads were aligned, and the APA site usage was determined by using the method described above.

Variant design and analysis code are available at https://github.com/MaxChien1996/replace_UG_in_first_800_bp_of_ELK1_extended_3_prime_UTR.

### SH-SY5Y and SK-N-BE(2) TDP-43 knockdown for RNA-seq

SH-SY5Y and SK-N-BE(2) cells were transduced with a SMARTvector lentivirus (V3IHSHEG_6494503) containing a doxycycline-inducible shRNA cassette for TDP-43. Transduced cells were selected with puromycin (1 μg ml^−1^) for 1 week, before being plated as single cells and expanded to obtain a clonal population. Cells were grown in DMEM/F12 + GlutaMAX (Thermo Fisher Scientific) supplemented with 10% FBS (Thermo Fisher Scientific) and 1% penicillin−streptomycin (Thermo Fisher Scientific). For induction of shRNA against TDP-43, cells were treated with the following amounts of doxycycline hyclate (Sigma-Aldrich) and collected after 10 days:

- For experiments in SH-SY5Y cells (curves), 75 ng ml^−1^
- For experiments in SH-SY5Y cells (cycloheximide), 25 ng ml^−1^
- For experiments in SK-N-BE(2) cells, 1,000 ng ml^−1^

### RNA-seq

Strand-specific, poly(A)-enriched sequencing libraries for the ‘Humphrey i3 cortical’ dataset were prepared using the KAPA mRNA Hyper Prep Kit. One hundred total nanograms of RNA was used as input material for poly(A)-positive mRNA capture. Fragmentation was performed for 6 minutes at 85 °C to obtain a target fragment size of 300−400 bp, and 13 cycles of PCR amplification were performed. The resulting libraries were sequenced 2 × 150 bp on an Illumina NextSeq 2000 machine.

RNA was extracted from i3Neurons (‘Zanovello i3 Cortical’) and SH-SY5Y and SK-N-BE(2) cells using the RNeasy Mini Kit (Qiagen) following the manufacturer’s protocol including the on-column DNA digestion step. RNA concentrations were measured by NanoDrop, and 1,000 ng of RNA was used for reverse transcription. Samples undergoing RNA-seq were furthermore assessed for RNA quality on a TapeStation 4200 (Agilent), resulting in an RNA integrity number (RIN) higher than 9.4 for all samples. Sequencing libraries were prepared with poly(A) enrichment using the TruSeq Stranded mRNA Prep Kit (Illumina) and sequenced on an Illumina HiSeq 2500 or NovaSeq 6000 machine at UCL Genomics with the following specifics:

- SH-SY5Y cells: 2 × 100 bp, depth >40 million per sample
- SK-N-BE(2) and ‘Zanovello i3 Cortical’ cells: 2 × 150 bp, depth >40 million per sample

### RNA-seq data processing

‘Humphrey i3 Cortical’ samples were processed as previously described^69^ using the RAPiD-nf Nextflow pipeline. In brief, adapters were trimmed from raw reads using Trimmomatic^70^ version 0.36, and reads were aligned to the GRCh38 genome build using gene models from GENCODE version 30 (ref. ^71^) with STAR^72^ version 2.7.2a. The RAPiD-nf pipeline is available at https://github.com/CommonMindConsortium/RAPiD-nf/.

The ‘Brown’ SH-SY-5Y, SK-N-BE(2) and i3Neuron datasets were processed as previously described^10^. Unless otherwise stated, all short-read RNA-seq datasets were processed using the following pipeline. Raw reads in FASTQ format were quality trimmed for a minimum Phred score of 10 and otherwise default parameters using fastp^73^ (version 0.20.1). Quality trimmed reads were aligned to the GRCh38 genome build using gene models from GENCODE version 40 (ref. ^71^) with STAR^72^ (version 2.7.8a). Quality trimmed reads are used as input for any tools that require FASTQ files as input (for example, PAPA and Salmon). Our alignment pipeline is implemented in Snakemake^74^ and is available at https://github.com/frattalab/rna_seq_snakemake.

### SLAM-seq

SLAM-seq was performed on cortical-like i3Neurons following protocols adapted from Herzog et al.^51^. Samples were treated with 100 µM 4SU on day 7 for 0, 1, 4, 8, 12 and 24 hours before immediate wash with PBS. Each timepoint had two replicates for both control and TDP-43 knockdown, excluding 4 hours where one of the control replicates did not pass RNA quality controls and so was not submitted for sequencing.

RNA was extracted using the Qiagen RNA isolation and purification kit. RNA concentration was estimated using a NanoDrop Microvolume Spectrophotometer (Thermo Fisher Scientific). After ensuring an adequate amount of RNA in each sample, iodoacetamide (IAA) treatment was applied to each, facilitating the thiol modification of incorporated 4SU.

Sequencing libraries were prepared with the KAPA RiboErase RNA Hyper Kit and sequenced (2 × 250 bp) on an Illumina NovaSeq SP. Using the ‘rna_seq_snakemake’ alignment pipeline (https://github.com/frattalab/rna_seq_snakemake), raw FASTQ files were quality trimmed using fastp^73^ with the parameter ‘qualified_quality_phred: 10’ and aligned without soft clipping to the GRCh38 genome build using STAR^72^ (version 2.7.0f) with gene models from GENCODE version 34 (ref. ^71^). GRAND-SLAM (version 2.0.7b) was run on the aligned data using gene models from GENCODE version 34 (ref. ^71^) using the ‘-trim5p 10 -trim3p 10’ parameter to ignore mismatches at the ends of reads. The output files containing the estimated new-to-total RNA ratios (NTRs) of each gene were used to estimate the half-life of each gene using the recommended workflow in grandR^75^.

For analyses on specific isoform stability, the reads were aligned to a custom general transcription factor (GTF) containing all 3’UTR isoforms quantified by PAPA (see the ‘Identification of cryptic last exons with PAPA’ section) using the fastq2EZbakR pipeline (https://github.com/isaacvock/fastq2EZbakR, version 0.2.0). Half-lives for the bins aligning to the *ELK1* long and short UTR were calculated using the ‘EstimateFractions’ function from EZbakR^76^ version 0.0.0.9000 to retrieve the fraction of old RNA. Decay constants and 95% confidence intervals for each bin were calculated using a custom script (‘isoform_specific_analysis.Rmd’ in the ‘tdp43-apa’ repository) using weighted nonlinear regression. In brief, for each bin and condition, fraction old RNA estimates were inversely weighted proportional to the squared s.e. estimate, and nonlinear least-squares regression was performed to model the fraction remaining as an exponential decay function. We note that this method is used here to detect relative changes in RNA half-lives between conditions and not to provide the exact half-life estimates.

### PAPA—pipeline to detect cryptic last exons

Although there are many tools for de novo alternative polyadenylation detection within 3’UTRs from RNA-seq data, all suffer from poor performance with respect to matched 3’ end sequencing approaches^77,78^. These tools also cannot detect upstream poly(A) sites or define complete last exon structure. Aptardi is a deep-learning-based approach to refine predicted 3’ ends of reference or assembled transcriptomes^79^ but was excluded from a recent benchmarking study due to compute times and resource requirements^78^. TECtool (version 0.4) trains a machine learning model on annotated last exons to classify novel intronic last exons defined upstream of poly(A) sites from the PolyASite atlas^24^ but can only define ALEs and only supports single-end RNA-seq data, substantially impacting sensitivity. Inspired by findings that general purpose transcript assemblers can sufficiently define individual exons^80^ and a previous workflow combining matched short-read and 3’ enriched sequencing^18^, our approach extracts last exons from StringTie^19^ assembled transcripts and filters based on proximity to 3’ end sequencing-derived poly(A) sites. Additionally, we rescue events with poly(A) signal hexamers near the 3’ end, an important feature in discriminating 3’UTRs from other transcriptomic regions^28^ that can also mitigate incomplete coverage of cellular contexts and experimental conditions by 3’ sequencing databases.

### Pipeline setup

Transcript assemblies for individual samples were generated using StringTie 2.1.7 (annotation-guided mode). Grouping by experimental condition, a redundant assembly was generated using GffCompare^81^ 0.11.2. Next, condition-wise, transcript-level mean transcripts per million (TPMs) were calculated, assigning 0 TPM if absent in a sample. Transcripts were filtered for >1 mean TPM to improve global assembly accuracy^82^. Next, we extracted last exons from sample-wise assembled transcripts and identified novel events that satisfy the following criteria:

- Predicted PAS does not overlap annotated exons.
- ALEs—last intron is contained within annotated introns with exactly matching 5’ss
- IPAs—last exon overlaps annotated exon with a matching 5’ end (exact for internal exons, within 100 nucleotides (nt) for first exons due to known imprecision of assembled transcript start sites)
- 3’Ext—overlaps annotated last exon with exactly matching 5’ ends and extends the longest exon at the locus
- IPA and 3’Ext—extends annotated exon by minimum distance (default 100 nt)

Filtered novel last exons were then merged by condition into single GTFs to select a condition-wise representative prediction based on 3’ end precision. Last exon 3’ ends within 100 nt of PolyASite 2.0 database^20^ PASs were retained and updated to database coordinates. Alternatively, last exons containing any of the 18 poly(A) signal hexamers^21^ in the final 100 nt were retained, selecting the exons with hexamers closest to the expected 21-nt upstream position.

We then combined the filtered novel and annotated last exons into a combined transcriptome reference. We then defined ‘last exon identifiers’ based on overlapping regions. Overlapping last exons of each gene were assigned a common identifier, with 3’Exts receiving a unique identifier to the exons the annotated last exons they extend. Regions overlapping annotated first or internal exons were removed to retain only unique last exon sequences. Last exons with 3’ ends overlapping annotated first/internal exons were excluded.

Transcript sequences were extracted using GffRead^81^ 0.12.1 and used to construct a decoy-aware transcriptome index using Salmon^22^ 1.5.2 (GRCh38 genome build as decoys). Samples were subsequently quantified using Salmon^22^ 1.5.2 (‘–gcBias’ and ‘–seqBias’ flags enabled). TPM values were summed by the last exon identifier, and estimated counts were generated with tximport^83^ 1.26.0 (‘countsFromAbundance=lengthScaledTPM’) for differential isoform usage testing with DEXSeq^23^ 1.44.0. PAS usage was calculated by dividing last exon isoform expression (TPM) by total gene isoform expression.

PAPA 0.2.0, available at https://github.com/frattalab/PAPA, is implemented as a Snakemake^74^ pipeline using PyRanges^84^ 0.0.115 for interval operations and pyfaidx^85^ 0.6.2 and BioPython^86^ 1.79 for genomic sequence operations. Conda environments are used for dependency management.

### Identification of cryptic last exons with PAPA

We ran PAPA in ‘identification’ mode to predict novel last exons in the i3Neuron, ‘Zanovello’ SH-SY5Y and SK-N-BE(2) datasets. We provided GENCODE version 40 (ref. ^71^) annotations filtered for protein-coding and lncRNA gene transcripts with a ‘transcript support level’ value ≤ 3 and without the ‘mRNA_end_NF’ tag^87^.

Predicted last exon GTF files were combined into a single GTF using PAPA’s ‘combine_novel_last_exons.py’ script. All datasets were then quantified and assessed for differential usage using a unified transcriptome reference combining novel and annotated last exons from the filtered GTF. Differential usage was performed using the standard DEXSeq workflow, with the differentiation date added as a covariate for the ‘Klim i3 motor’ dataset^40^. We defined cryptic APAs as DEXSeq adjusted *P* < 0.05, mean control usage < 10% and change in mean usage > 10% (TDP-43 knockdown, control). We further manually curated cryptic IPAs, as manual inspection suggested frequent artifacts at regions of reduced coverage in intron retention loci.

### Cryptic PAS validation using PATRs

TDP-43 knockdown samples from all in vitro datasets were used. Soft-clipped alignments were extracted and 3’ ends inferred based on the strandedness of the RNA-seq protocol (reads with soft clips at both ends were excluded from unstranded protocols). PATRs were defined as soft-clipped regions ≥6-nt with ≥80% tail nucleotide content^30^ (A for rightmost/plus strand; T for leftmost/minus strand) or 3−5-nt overhangs with 100% tail content, with the 3’ most-aligned coordinate defining the putative PAS.

PATRs were pooled across datasets and clustered using an iterative approach approximating PolyASite’s algorithm^20^. PASs were extended ±12 nt and overlapped, selecting the position with highest read support as representative. Reads within 12 nt of the representative coordinate were collapsed into a cluster, with the process repeated until all PATRs were assigned.

For cryptic PAS validation, we generated 1,000 covariate-matched annotated PAS samples by stratified sampling without replacement using ‘matchRanges’ from nullranges^88^ version 1.8.0. We matched for expression (log_2_(median TPM + 1)) and the number of unique PASs (separated by ≥12 nt), assessing covariate balance using the ‘bal.tab’ method from cobalt^89^ version 4.5.5.

We then computed distances between annotated/cryptic PASs and nearest PATR clusters, assigning 0 for overlaps. We reported overlap if one or more PASs passed the distance threshold (10, 25, 50, 100 and 200 nt). At each threshold, we computed a group-wise fraction of overlapping events ($\widehat{{p}_{i}}$) and computed two-sided empirical *P* values to assess whether cryptic and annotated PASs arose from the same distribution as follows:

$$p=\frac{1}{N}\mathop{\sum }\limits_{i=1}^{N}I(|{\hat{p}}_{i}-\hat{\mu }|\ge |\widehat{{p}_{\rm{obs}}}-\hat{\mu }|)$$

where *N* = 1,000 (total annotated samples), $\hat{\mu }$ = annotated distribution mean $\widehat{{p}_{i}}$, $\widehat{{p}_{\rm{obs}}}$ = cryptic PAS $\widehat{{p}_{i}}$ and I(.) is an indicator function.

The PATR extraction pipeline, available at https://github.com/SamBryce-Smith/bulk_polyatail_reads (version 0.1.0), is implemented using Snakemake^76^ version 7.32.4, Python 3.10.13, PyRanges^86^ version 0.0.129, pysam version 0.22.0, pandas version 2.1.4, NumPy version 1.26.3, pyarrow version 15.0.0 and fastparquet version 2024.2.0. Cryptic PAS validation scripts are available under the ‘preprocessing’ directory at https://github.com/frattalab/tdp43-apa/.

### DaPars2 comparison

Transcript models for *ELK1*, *SIX3* and *TLX1* were extracted from National Center for Biotechnology Information RefSeq version 110 annotation. 3’UTR and last exons were overlapped with 3’Ext intervals. If any overlap was detected, the 3’ end coordinate of the annotated interval was updated to the 3’Ext 3’ end. Upstream transcript intervals were otherwise unmodified. We then analyzed the ‘Seddighi i3 Cortical’ dataset with DaPars2 (ref. ^31^) using a Snakemake pipeline developed for the APAeval project^78^ (available at https://github.com/iRNA-COSI/APAeval). Two separate runs with the original or updated transcript models were performed. BED files of predicted PASs and their relative usages parsed from the DaPars2 output file were used for downstream analysis, extracting the distal events to represent cryptic 3’Ext predictions.

### TDP-43 iCLIP analysis

The SH-SY5Y TDP-43 iCLIP data (ArrayExpress: E-MTAB-11243) were generated and processed as previously described^10^. iCLIP peaks from the two independent replicates were merged into non-redundant intervals for all subsequent analysis.

Cryptic events were defined as last exon isoforms passing cryptic thresholds in any in vitro dataset. The probability of detecting TDP-43 binding events via iCLIP is influenced by the abundance of target RNAs, but, by pooling cryptic events across datasets, we cannot control for the confounding influence of RNA expression between groups. We, therefore, defined background events as isoforms that were assessed for differential usage in all SH-SY5Y datasets and had an adjusted *P* > 0.05 across all datasets, which biases against observing enriched binding in the cryptic group.

For 3’Ext events, the most distal annotated poly(A) site is selected to represent the proximal site, and background events represent loci with a predicted novel 3’UTR extension. For other event categories, background events include annotated and novel events. Our approach to define a common last exon reference across datasets can result in non-redundant intervals being predicted for the same last exon isoform. We, therefore, implemented a collapsing strategy to define a single representative interval for each event.

First, we filtered for novel predictions matching a PolyASite reference PAS. If distinct reference PASs are reported for the same isoform, the site predicted in the most independent datasets is selected as representative. If distinct sites are detected in the same number of independent datasets, the most proximal site is arbitrarily selected. PolyASite PAS intervals represent clusters. If distinct 3’ end predictions overlap with the same PAS cluster, the prediction closest to the PolyASite representative coordinate is selected (most distal prediction is arbitrarily selected in case of ties).

If no isoforms matched a PolyASite PAS, we selected a representative prediction whose poly(A) signal motif minimizes the deviance from the characteristic position 21 nt upstream of the PAS. In case of ties, the most proximal prediction was arbitrarily selected. As distinct intervals still remained for background ALEs and IPAs after 3’ end collapsing, we arbitrarily selected the most distal 3’ end for nine background IPAs and the most proximal 5’ end for four background ALEs.

We constructed TDP-43 binding metaprofiles by extending genomic landmarks by 500 nt in both directions and computing per-position coverage by iCLIP peaks using BEDTools^90^ version 2.31.0. We then calculated mean coverage (fraction of events with an overlapping peak) and s.e. for each position relative to the landmark. We plotted LOESS-smoothed (‘span’ = 0.1) coverage and confidence intervals (±1 s.e.).

### De novo motif enrichment analysis

To perform de novo motif enrichment, we adapted PEKA^32^, which identifies kmers with positional enrichment at iCLIP peaks relative to background crosslink sites while normalizing to the general occurrence in the surrounding genomic context. Therefore, we can substitute iCLIP peaks and global crosslink sites for cryptic and background landmarks, respectively, to identify positionally enriched kmers with respect to cryptic landmarks. For all comparisons, we ran PEKA to search for enriched 6-mers in the proximal window of interest set to 250 nt (the broad window in which iCLIP peaks were observed), and the distal window was set to 500 nt (to maintain consistency with the overall search space for iCLIP peaks). The ‘percentile’ flag was set to 0 to switch off thresholding of background regions based on read count, and the ‘relpos’ flag was set to 0 to consider all positions in the proximal window when calculating the enrichment score.

Preferred TDP-43 binding 6-mers were extracted from Halleger et al.^6^. In brief, the 6-mers were defined using PEKA as the top 20 most enriched kmers around intronic iCLIP crosslinks across all wild-type, A326P, G294A, G335A, M337P and Q331K and a 316del346 GFP−TDP-43 in HEK293 cells. The 20 were subsequently separated into the following three groups based on a gradient of enrichment in wild-type and G335A TDP-43 with respect to A326 and 316del346 variants and their consensus sequence:

- YG-containing [UG]n 6-mers: UGUGUG, GUGUGU, UGUGCG, UGCGUG, CGUGUG, GUGUGC
- YA-containing [UG]n 6-mers: AUGUGU, GUAUGU, GUGUAU, UGUGUA, UGUAUG, UGCAUG
- AA-containing [UG]n 6-mers: GUGUGA, AAUGAA, GAAUGA, UGAAUG, AUGAAU, GUGAAU, GAAUGU, UUGAAU

where ‘Y’ corresponds to a pyrimidine nucleotide. To assess their overrepresentation among enriched 6-mers relative to cryptic landmarks, we performed a one-sided GSEA using fgsea^42^ version 1.24.0 with default settings for each cryptic landmark. The three 6-mer groups and the union of all three groups were provided as input pathways, and kmers were ranked by their PEKA score. After independent runs for each landmark, Benjamini−Hochberg adjusted *P* values were calculated with respect to all tested landmarks and 6-mer sets and used to evaluate statistical significance.

To generate maps of coverage of specific kmers, we used cv_coverage^91^ version 1.1.0 (https://github.com/ulelab/cv_coverage) to scan for occurrences of the YG-containing [UG]n 6-mers in a 500-nt window around cryptic and background landmarks, disabling weighting the occurrence by cDNA count. For coverage plots, the percentage occurrences of each 6-mer were summed separately for the cryptic and background regions. The percentage occurrences were converted to mean coverages and visualized as described for iCLIP maps.

The adapted PEKA code is available at the ‘output_mods’ branch of the following forked copy of the PEKA repository: https://github.com/SamBryce-Smith/peka. A Snakemake pipeline to run PEKA and cv_coverage is available in the ‘motifs/peka_snakemake’ directory of the ‘tdp43-apa’ repository.

### Postmortem RNA-seq analysis—FACS-seq data processing

Sequenced reads from FACS-sorted frontal cortex neuronal nuclei^34^ were processed as described in Brown et al.^10^. The data are available in the Gene Expression Omnibus (GEO) at GSE126543.

### Quantification of cryptic last exons in postmortem FACS-seq data

Nuclear RNA-seq libraries contain both nascent and processed RNA. We, therefore, constructed decoy transcript models that reflect alternative processing decisions at ALE and IPA loci (for example, intron retention) to limit the confounding effect of nascent RNAs on transcript quantification^22^.

First, we extracted cryptic ALE and IPA coordinates from the unified transcript reference used to quantify cell culture datasets. We then generated decoy transcript models separately for each event type. For IPA events, the unique cryptic IPA region was extended to incorporate the adjacent upstream annotated internal exon. Then, a ‘spliced’ decoy transcript that traverses the annotated internal exon to the downstream annotated internal exon was generated, alongside an ‘intron retention’ decoy transcript that contains the same pairs of internal exons merged with the intervening intron. For ALEs, a ‘retained intron’ decoy transcript was generated that corresponds to the complete intronic region in which the ALE is contained. No decoy transcript models were generated for 3’Ext, 3’shortening and ‘complex’ events or for ALEs that are the most distal annotated isoform of their gene. Decoy transcript and gene identifiers were appended with suffixes to differentiate from cryptic APAs and annotated transcripts. Finally, the decoy transcripts and cryptic APAs were returned to the unified transcript reference to generate a decoy-augmented last exon reference for quantification.

The decoy-augmented reference was quantified with Salmon version 1.8.0 (ref. ^22^) using the ‘salmon’ sub-pipeline available at https://github.com/frattalab/rna_seq_single_steps. As with PAPA, samples are quantified against a decoy-aware transcriptome index with full genome sequence (GRCh38 build) used as decoys^92^ and the ‘–gcBias’ and ‘–seqBias’ flags enabled.

Calculation of percent poly(A) usage (PPAU) was performed using a copy of the ‘tx_to_polyA_quant.R’ script from the PAPA repository. Sample-wise differences in PPAU were calculated by subtracting PPAU in the TDP-43-positive population from the TDP-43-negative population (that is, a positive difference indicates enrichment in the TDP-43-depleted population). Cryptic APAs with a median sample-wise enrichment of more than 5% were considered as enriched. Scripts to construct decoy transcripts and analyze quantifications are available under the ‘postmortem’ subdirectory at https://github.com/frattalab/tdp43-apa.

### NYGC RNA-seq data

The sequencing libraries were generated^35,93^ and processed^13^ as previously described. Samples were classified into disease subtypes as previously described^13^. In brief, FTD subtypes were classified by pathology according to the presence of TDP-43 inclusions (FTLD-TDP), FUS or Tau aggregates. Patients with ALS were subcategorized based on presence (ALS-non-TDP) or absence (ALS-TDP) of reported SOD1 or FUS mutations. The following samples were considered as regions where TDP-43 pathology (and specific cryptic junction expression) is expected: motor (ALS-TDP), frontal and temporal cortex samples (FTLD-TDP and ALS-TDP) and cervical, lumbar and thoracic spinal cord samples (ALS-TDP).

We opted to quantify ALE events using junction reads, which provide direct quantification of the occurrence of a splicing event. As of version 0.2, PAPA does not directly report splice junctions associated with ALE events. However, as the filtering criteria applied by PAPA require putative ALE events to have a terminal splice junction with a direct match to an annotated 5’ss, it is possible to infer splice junctions from reference annotation using just the reported last exon coordinates. For ALEs fully contained within annotated introns, the splice junction is defined from the intron start to the start of the ALE. If last exons are distal to the annotated gene, then the closest upstream annotated intron is found. The splice junction is subsequently defined as the region from the intron start to the start of the ALE. Finally, for annotated ALEs, all annotated introns that terminate at the ALE are reported as splice junctions for the event. The above steps are implemented in a custom script, ‘last_exons_to_sj.py’, available at the ‘tdp43-apa’ GitHub repository.

Splice junctions for cryptic ALEs and cryptic splice junctions identified in cortical-like i3Neurons^13^ were quantified across the NYGC RNA-seq cohort by extracting counts for provided junctions from the ‘.SJ.out.tab’ files produced by STAR^72^. The code is implemented in the ‘bedops_parse_star_junctions’ version 0.1.0 Snakemake pipeline and is available at https://github.com/SamBryce-Smith/bedops_parse_star_junctions.

We defined detection criteria to prioritize cryptic splice junctions that are specifically in tissue types and samples with expected TDP-43 pathology. Junctions are considered expressed if at least two spliced reads are detected in a sample. Junctions are considered selectively expressed if expressed in at most 0.5% of all samples where TDP-43 pathology is not expected and in at least 1% of samples where TDP-43 pathology is expected. We note that such criteria will exclude events with enriched expression in tissues with expected TDP-43 proteinopathy but that have basal expression in unknown cell types not represented in our in vitro compendium. Such events may still have relevance in mechanisms of disease in specific cell types but are less suitable for discriminating samples with TDP-43 proteinopathy.

### Ribo-seq analysis

i3Neuron Ribo-seq data were generated and processed as previously described^13^. Uniquely mapped reads were assigned to genes based on the union of annotated ‘CDS’ entries in the GENCODE version 34 standard annotation released using featureCounts^94^ version 2.0.1. Differential expression between TDP-43 knockdown and control was performed using DESeq2 (ref. ^95^) version 1.38.3, and differentially translated genes were defined based on a Benjamini−Hochberg adjusted *P* value threshold of 0.05. Any last exon passing our cryptic criteria in at least one of the i3 Neuron datasets (Brown i3 cortical, Seddighi i3 cortical, Humphrey i3 cortical) was considered for intersection with differentially translated genes.

GSEA was performed using fgsea^42^ version 1.24.0 with default settings. Cryptic 3’Ext, IPA and ALE containing genes were provided as input pathways, and moderated fold changes were calculated with the ‘lfcShrink’ function from the DESeq2 package using the default apeglm^96^ method as the shrinkage estimator to rank genes. A threshold of 0.05 Benjamini−Hochberg adjusted *P* value was used to determine statistical significance.

Read counting was performed using the ‘feature_counts’ sub-pipeline available at https://github.com/frattalab/rna_seq_single_steps. Custom scripts used to perform differential expression and pathway analysis are available at https://github.com/frattalab/tdp43-apa.

For cross-referencing with differential RNA expression, we used differential expression analysis from cortical-like i3Neurons performed as previously described^13^. Cryptic last exon-containing genes were highlighted if they passed the statistical significance threshold in the Ribo-seq differential expression analysis.

### Analysis of ELK1 transcription factor activity

ELK1 target genes in HeLa cells were accessed from the ChIP-Atlas^97^ on 15 November 2023. We used the ‘Target genes’ module to obtain a list of target genes that have a ChIP–seq peak within ±1 kb of transcription start sites. The resulting list contained two HeLa datasets (GSM608163 and GSM935326) and was filtered to target genes identified in both datasets. Given a reported redundancy of function between ELK1 and other members of the TCF family^98^ (ELK3 and, particularly, ELK4), we also attempted to define a unique set of ELK1 target genes. ELK4 target genes in HeLA cells were accessed from ChIP-Atlas on 29 November 2023 using the same parameters. The resulting list contained three HeLa datasets (GSM608161, GSM608162 and GSM935351), and we again filtered for target genes identified in all datasets. ELK3 HeLa ChIP–seq data were not available through ChIP-Atlas at the time of publication and were not considered for further redundancy. ELK3 RNA levels are 10× lower than ELK3 and ELK4 in HeLa TDP-43 knockout cells^49^, so we anticipate that this is unlikely to affect our conclusions. ELK1 and ELK4 target gene lists were intersected to define common and unique target genes for each transcription factor. Final target gene lists used are reported in Supplementary Table 5.

RNA-seq data from HeLa cells with TDP-43 knockout^49^ were accessed from GSE136366. The data were processed and differential expression was performed as previously described^10^. Genes were ranked by DESeq2’s test statistic (log_2_ transformed fold change divided by the s.e. of the fold change) after removing genes with differential splicing upon TDP-43 knockout, where we can expect to attribute any changes in gene expression to TDP-43 loss of function. Differentially spliced genes were defined using MAJIQ^99^, considering any genes with a probability greater than 0.95 as differentially spliced. The target gene sets described above were used as input pathways to fgsea^42^ version 1.24.0 using default settings.

### Subcellular Frac-seq analysis

The neural progenitor cell short-read Frac-seq data^56^ were downloaded from the GEO at accession number GSE244655. RNA-seq quality control and processing was performed as previously described (see ‘RNA-seq data processing’ section). The PAPA index was used to quantify ELK1 isoform expression with Salmon version 1.8.0, using the ‘salmon’ sub-pipeline available at https://github.com/frattalab/rna_seq_single_steps. TPM values for the ELK1 3’Ext were pooled across ribosome-associated fractions (monosome, light polysome and heavy polysome), and PPAU was recalculated for each fraction and replicate. All ELK1 3’Ext PPAU values were then normalized to the cytosol PPAU within each replicate for subsequent visualization. Statistical significance was evaluated using a two-sided one-sample *t*-test after log transforming the PPAU ratios, testing the null hypothesis that the mean is equal to log(1).

### Statistics and reproducibility

Our study design involved multiple stages. First, we used transcriptome-wide hypothesis testing of high-throughput RNA-seq datasets to identify a panel of TDP-43-sensitive cryptic polyadenylation events. We performed this screen in neuronal cell models, where we could reliably deplete TDP-43 levels to mimic nuclear loss in disease. We then screened this panel in specialized and bulk postmortem tissue datasets to highlight events whose expression patterns were consistent with disease and TDP-43 pathology status. Finally, we performed targeted experimental assays to validate observations from high-throughput sequencing and to investigate the molecular consequences of specific cryptic polyadenylation events.

Sample sizes for postmortem tissue analysis (Fig. 2b) were determined by the availability of samples at the time of analysis. Sample size for the NYGC ALS Consortium was determined by the number of available samples at the time of analysis (corresponding to a subset of the 21 February 2023 data freeze) as data collection is still ongoing. Sample sizes for novel omics datasets and experimental validation were determined based on previous studies succeeding with similar aims to identify novel isoforms, perform targeted validation and assess their downstream effects on RNA and protein expression^10^.

All statistical tests were performed two-sided. One-sample *t*-tests were performed using log-transformed ratios of within-replicate, control-normalized values (mean count for FISH experiments and percent PAS usage for Frac-seq). Log transformation is a standard transformation to bring a distribution closer to a normal distribution, but the assumption of normally distributed transformed data was not formally tested. For Student’s unpaired *t*-test (3’RACE experiments), equal variances were assumed, and the data distribution was assumed to be normal, but this was not formally tested. Unless otherwise stated, the Benjamini−Hochberg multiple-testing correction method was used to compute ‘adjusted’ *P* values.

Randomization was not used in this study, as most of the analyses (experimental and omics-based) were carried out in cell lines that are inherently homogenous. Randomization was not applicable in postmortem analyses as the variable of interest (disease status and expected TDP-43 pathology) is an observed variable, and no intervention was performed. No data were excluded from analysis. FISH images were analyzed blinded to TDP-43 depletion status. For all other experiments, the investigators were not blinded to experimental condition or disease status during experimentation and analysis.

### Reporting summary

Further information on research design is available in the Nature Portfolio Reporting Summary linked to this article.

## Online content

Any methods, additional references, Nature Portfolio reporting summaries, source data, extended data, supplementary information, acknowledgements, peer review information; details of author contributions and competing interests; and statements of data and code availability are available at 10.1038/s41593-025-02050-w.

## Supplementary information

Supplementary Information Supplementary Figs. 1−9

Reporting Summary

Supplementary Tables 1−9 Supplementary Tables 1−9 and supplementary table descriptions

Supplementary Data 1 Source Data for Supplementary Fig. 7

## Source data

Source Data Fig. 2 Statistical Source Data for Fig. 2b

Source Data Fig. 3 Unprocessed, uncropped western blots for Fig. 3c (page 1: Halo-tagged TDP-43; page 2: ELK1; page 3: Tubulin)

Source Data Fig. 3 Statistical Source Data for Fig. 3h

Source Data Extended Data Fig./Table 1 Statistical Source Data for Extended Data Fig. 1

Source Data Extended Data Fig./Table 4 Unprocessed, uncropped western blots (Extended Data Fig. 4a)

Source Data Extended Data Fig./Table 4 Statistical Source Data for Extended Data Fig. 4b−d

## Data Availability

This study analyzes existing and newly generated datasets. All existing datasets are publicly available from the accessions reported below. ‘Brown’ i3Neuron, SH-SY5Y and SK-N-BE(2) datasets are available through the European Nucleotide Archive (ENA) under accession PRJEB42763. The SH-SY5Y TDP-43 iCLIP data are available at the ENA under accession PRJEB49480 or at ArrayExpress under accession E-MTAB-11243. ‘Seddighi’ i3Neuron RNA-seq, i3Neuron nanopore direct RNA-seq and i3Neuron Ribo-seq data can be accessed at the Alzheimer’s Disease Workbench: https://fair.addi.ad-datainitiative.org/#/data/datasets/mis_spliced_transcripts_generate_de_novo_proteins_in_tdp_43_related_als_ftd_00005. The HeLa TDP-43 knockout (GSE136366), the FACS-sorted frontal cortex neuronal nuclei (GSE126543) and the ‘Klim’ iPSC-derived motor neurons (GSE12156) can be accessed at the GEO. Raw ChIP–seq data for ELK1 (GSM608163 and GSM935326) and ELK4 (GSM608161, GSM608162 and GSM935351) in HeLa cells can also be accessed through the GEO or in processed format as used in this study via ChIP-Atlas (https://chip-atlas.org/). The short-read neural progenitor cell Frac-seq data^56^ were downloaded from the GEO at accession GSE244655. RNA-seq data generated by the NYGC ALS Consortium and used in this study can be accessed through the GEO (GSE137810, GSE124439, GSE116622 and GSE153960). To request immediate access to new and ongoing data generated by the NYGC ALS Consortium and for samples provided through the Target ALS Postmortem Core, a genetic data request form can be completed at ALSData@nygenome.org. All sequencing datasets generated in this study have been deposited at the GEO: ‘Zanovello i3Neuron’ (GSE296710), ‘Humphrey i3Neuron’ (GSE296714), ‘Zanovello SH-SY5Y CHX’ (GSE296713), ‘Zanovello SH-SY5Y curve’ (GSE296712), ‘Zanovello SK-N-BE(2) curve’ (GSE296711) and i3Neuron SLAM-seq (GSE296716). An archive of minimal processed data required to reproduce analysis and figures presented in this paper is available from Zenodo^100^ (10.5281/zenodo.15538002). Source data are provided with this paper.

## Code availability

All visualization and statistical testing were performed in R^101^ version 4.3.2 using ggplot2 (ref. ^102^) version 3.4.4, ggpubr^103^ version 0.6.0, ggprism^104^ version 1.0.4 and ggrepel^105^ version 0.9.4 packages. Preprocessing for visualization and generation of supplementary tables was performed using tidyverse^106^ version 2.0.0, writexl^107^ 1.4.2 and data.table^108^ version 1.14. Unless otherwise stated, analyses requiring genomic interval operations or queries with bioinformatics data formats were performed in Python 3.10.11 using PyRanges^84^ 0.0.127, pandas^109^ version 2.0.2 and NumPy^110^ version 1.23.

All custom analysis code can be accessed at GitHub with specific versions archived at Zenodo. Alternative repositories for specific analyses are reported below and in the relevant Methods sections. Analysis and visualization code, along with conda^111^ and renv^112^ environments for dependency management, can be accessed at https://github.com/frattalab/tdp43-apa (10.5281/zenodo.15210472). The ‘salmon’ ‘feature_counts’ pipelines are available at https://github.com/frattalab/rna_seq_single_steps (10.5281/zenodo.15210438). The splice junction counting pipeline is available at https://github.com/SamBryce-Smith/bedops_parse_star_junctions (10.5281/zenodo.15209898). The PAPA Snakemake pipeline is available at https://github.com/frattalab/PAPA (10.5281/zenodo.15210362). The poly(A)-tail-containing read extraction Snakemake pipeline is available at https://github.com/SamBryce-Smith/bulk_polyatail_reads (10.5281/zenodo.15210306). The code for ELK1 3’UTR reporter design and analysis is available at https://github.com/MaxChien1996/replace_UG_in_first_800_bp_of_ELK1_extended_3_prime_UTR (10.5281/zenodo.15413618). The Snakemake RNA-seq processing and alignment pipeline is available at https://github.com/frattalab/rna_seq_snakemake (10.5281/zenodo.15463283).
