# Supplementary material for: TDP-43 loss induces cryptic polyadenylation in ALS/FTD
Source: Nat Neurosci. 2025 Oct 21;28(11):2190–200. doi: 10.1038/s41593-025-02050-w (PMC12586162; doi:10.1038/s41593-025-02050-w)
Supplement: Supplementary file 1 — Supplementary Figs. 1−9 [file 41593_2025_2050_MOESM1_ESM.pdf]

nature neuroscience

Supplementary information https://doi.org/10.1038/s41593-025-02050-w

TDP-43 loss induces cryptic polyadenylation in ALS/FTD

In the format provided by the authors and unedited

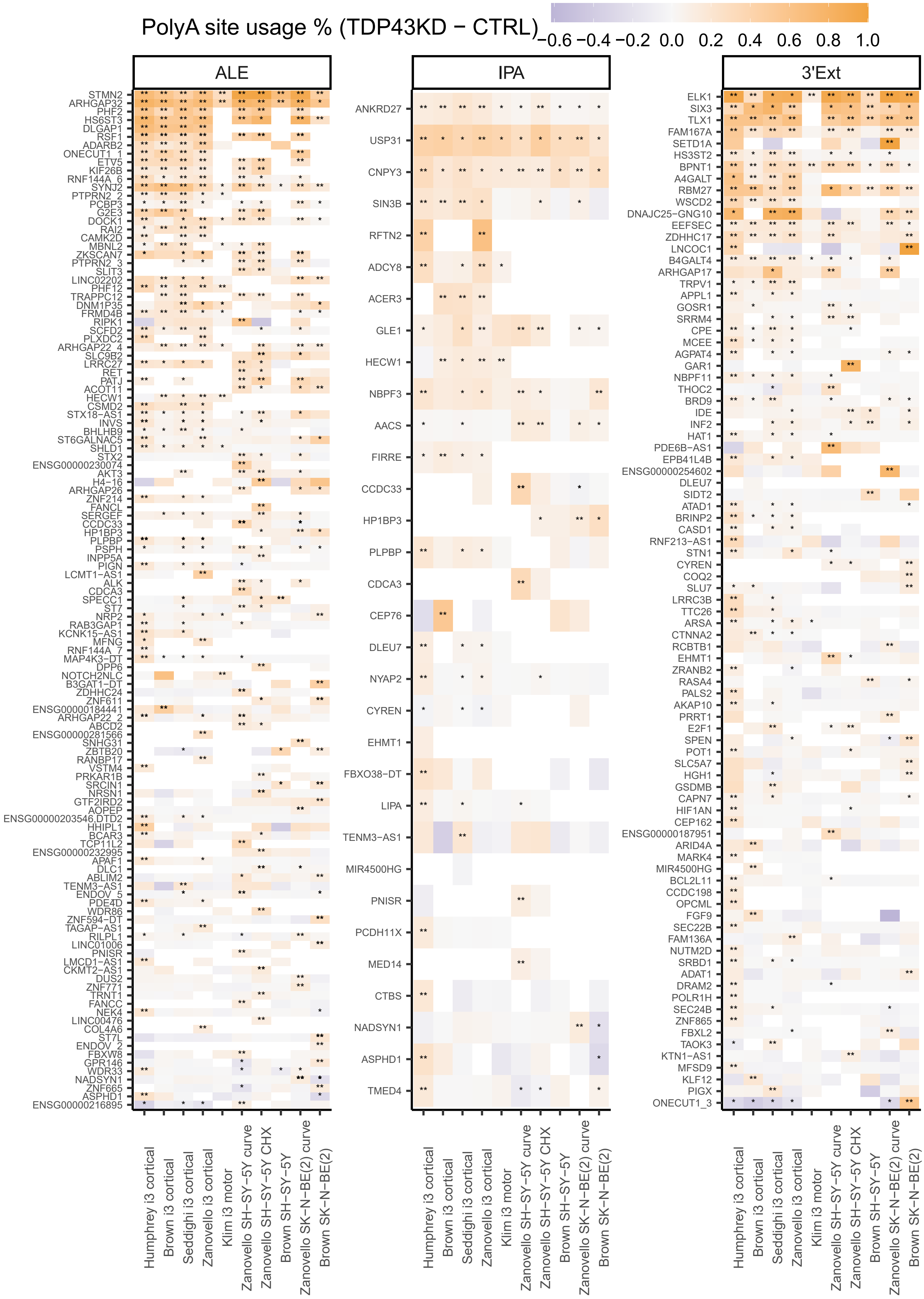

Supplementary Figure 1

Supplementary Figure 1 - Consistency of response to TDP-43 depletion across compendium of in-vitro datasets

Differential usage of cryptic APA events across the compendium of in-vitro datasets. Cells are coloured in accordance to magnitude and direction of change in usage, where positive values (orange) indicate increased usage in TDP-43 knockdown ('TDP43KD') samples. Blank cells indicate the event was not expressed at sufficient levels to be assessed for differential usage. Rows are sorted in decreasing order of the sum of $-\log_{10}$ transformed p-values weighted by the change in usage between TDP-43 knockdown and control samples (TDP43KD - CTRL) in each dataset. A single asterisk indicates that the isoform was considered significantly regulated in a dataset (Benjamini-Hochberg adjusted p-value < 0.05), and two asterisks indicate the isoform is considered cryptic in a given dataset.

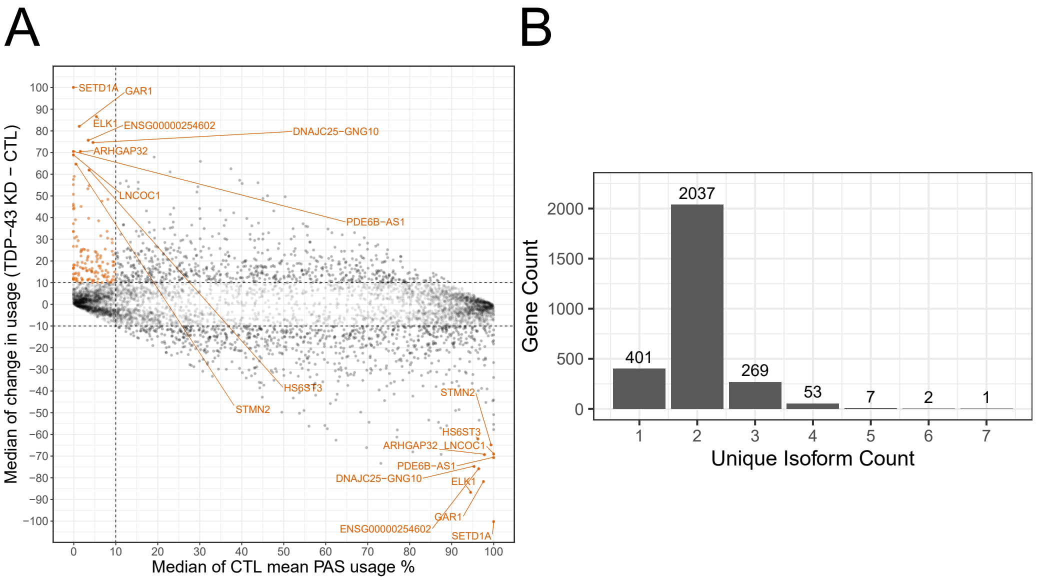

Supplementary Figure 2

Supplementary Figure 2 - Mirrored visualisation artefacts arising from visualising all regulated events in a gene

A) ‘Mirrored effect’ resulting from the visualisation approach used in Fig 1B. The 10 largest effect size cryptic APA-containing genes are highlighted in (A), and the reduction in their counterpart PASs are also shown.
B) Unique isoform counts for APA-containing genes visualised in Fig. 1B and A). A mirrored effect is expected for 73% of genes (2037 out of 2770) with two isoforms passing the significance threshold

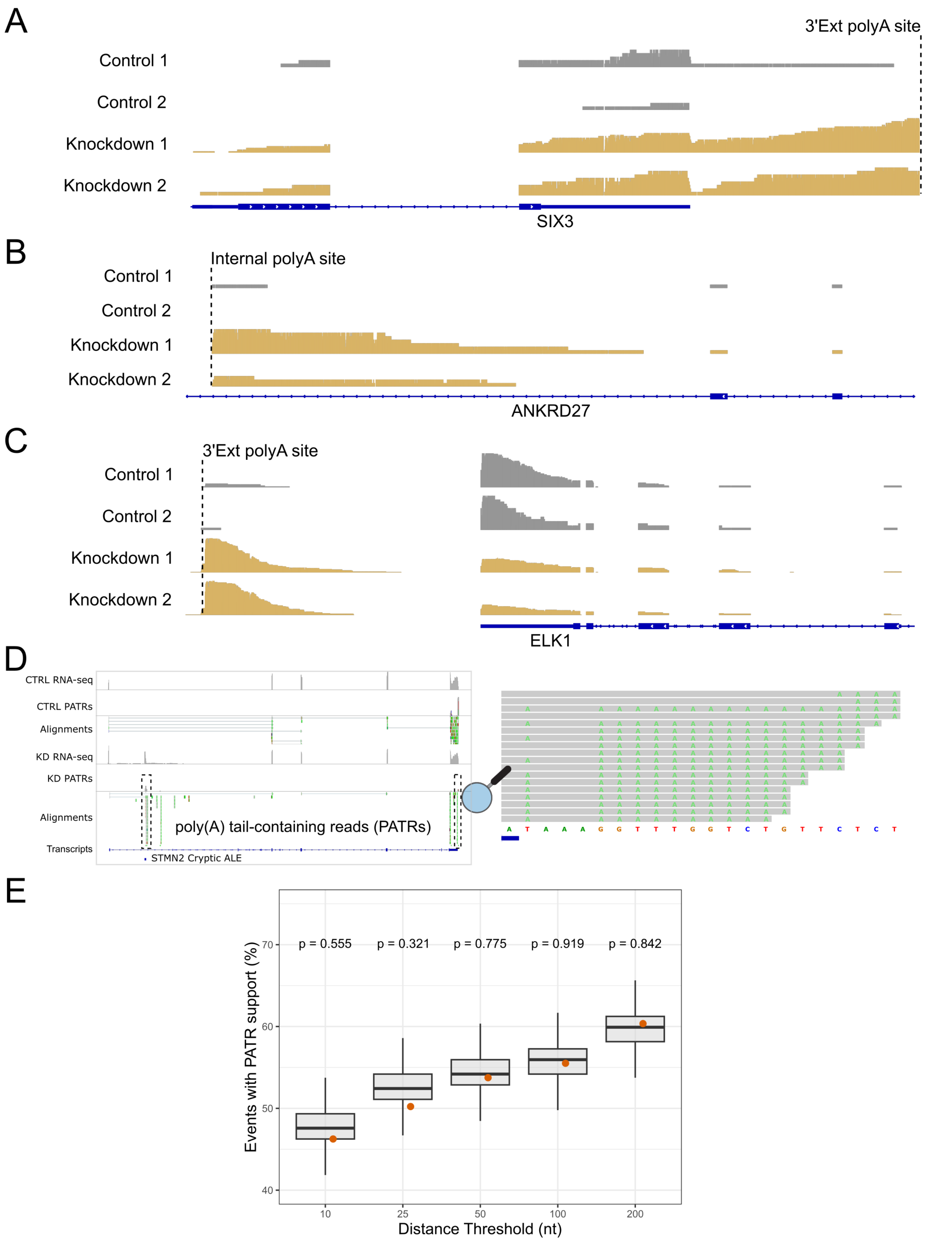

Supplementary Figure 3

Supplementary Figure 3 - The presence of cryptic APAs is validated by direct RNA Nanopore sequencing and the detection of poly(A)-tail containing RNA-seq reads (PATRs)

A) Direct RNA Nanopore sequencing traces from i3Neurons[1] confirm strong activation of the *SIX3* cryptic 3'Ext upon TDP-43 knockdown ('Knockdown') and near absent detection in controls. Numeric suffix corresponds to independent batch (1 control and 1 TDP-43 knockdown). Note that Direct RNA Nanopore sequencing is a ligation-dependent, primer-free method.
B) As in A), but for the *ANKRD27* IPA event (unsuccessful with 3'RACE).
C) As in A), but for the *ELK1* 3'Ext event.
D) IGV coverage trace for cryptic ALE-containing gene *STMN2* in i3Neurons ('Seddighi i3 cortical' dataset). All tracks correspond to RNA-seq reads pooled controls (CTRL) or TDP-43 knockdown (KD) samples. 'PATRs' refer to all putative PATRs (right-most soft-clip length = 3-5nt and 100 % As or >= 6nt and >=80% As) pooled across samples. (Right) Close-up view of putative PATR alignments (grey rectangles). Right-most consecutive runs of As represent the portion 'soft-clipped' regions of the alignment (part of the read sequence that does not align to the reference genome sequence below). The bottom blue box marks the annotated 3'end.
E) Overlap of cryptic PAS (orange) or covariate-matched annotated PAS sets (n=1000, grey box plot) with poly(A)-tail containing read (PATR) PAS clusters across a range of maximum distance thresholds (x-axis). The boxes correspond to the first, second and third quartiles and the whiskers span to the smallest and largest values within 1.5x the interquartile range. Two-sided empirical p-values at each distance threshold are presented without correction for multiple testing.

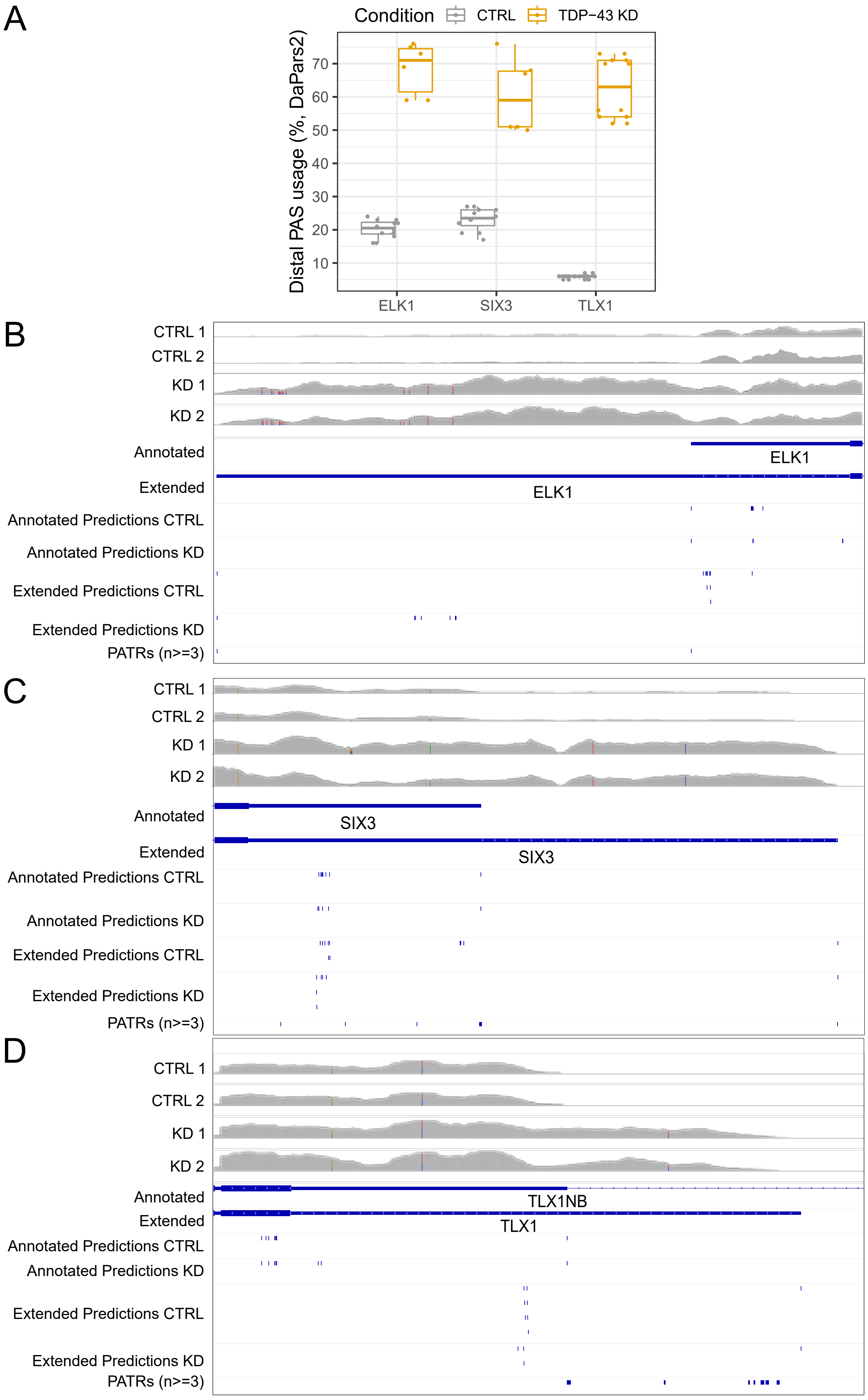

Supplementary Figure 4

Supplementary Figure 4 - DaPars2 identifies cryptic APA in *ELK1*, *SIX3* and *TLX1* when provided with 3'Ext coordinates.

C) Predicted distal PAS relative usage (%) in control (CTRL, grey) and TDP-43 knockdown (TDP-43 KD, orange) in the 'Seddighi i3 Cortical' dataset. Individual replicates represented as points. The boxes correspond to the first, second and third quartiles and the whiskers span from the smallest to the largest values.

D) DaPars2-predicted PAS locations in *ELK1* ('Seddighi i3 cortical' dataset). '*CTRL (1/2)*': control sample RNA-seq coverage traces; '*KD (1/2)*': TDP-43 knockdown sample RNA-seq traces; '*Annotated*': unmodified RefSeq v110 transcript models; '*Extended*': RefSeq v110 transcript models appended with 3'Ext coordinates; '*Annotated Predictions CTRL*': control sample PAS predictions when supplied with unmodified RefSeq v110 transcript models; '*Annotated Predictions KD*': TDP-43 knockdown sample PAS predictions when supplied with unmodified RefSeq v110 transcript models; '*Extended Predictions CTRL*': control sample PAS predictions when supplied with 3'Ext coordinates; '*Extended Predictions KD*': TDP-43 knockdown sample PAS predictions when supplied with 3'Ext coordinates; '*PATRs (n>=3)*' poly(A)-tail containing read PAS clusters supported by at least 3 reads.

E) As in B), but for *SIX3*.

F) As in B), but for *TLX1*.

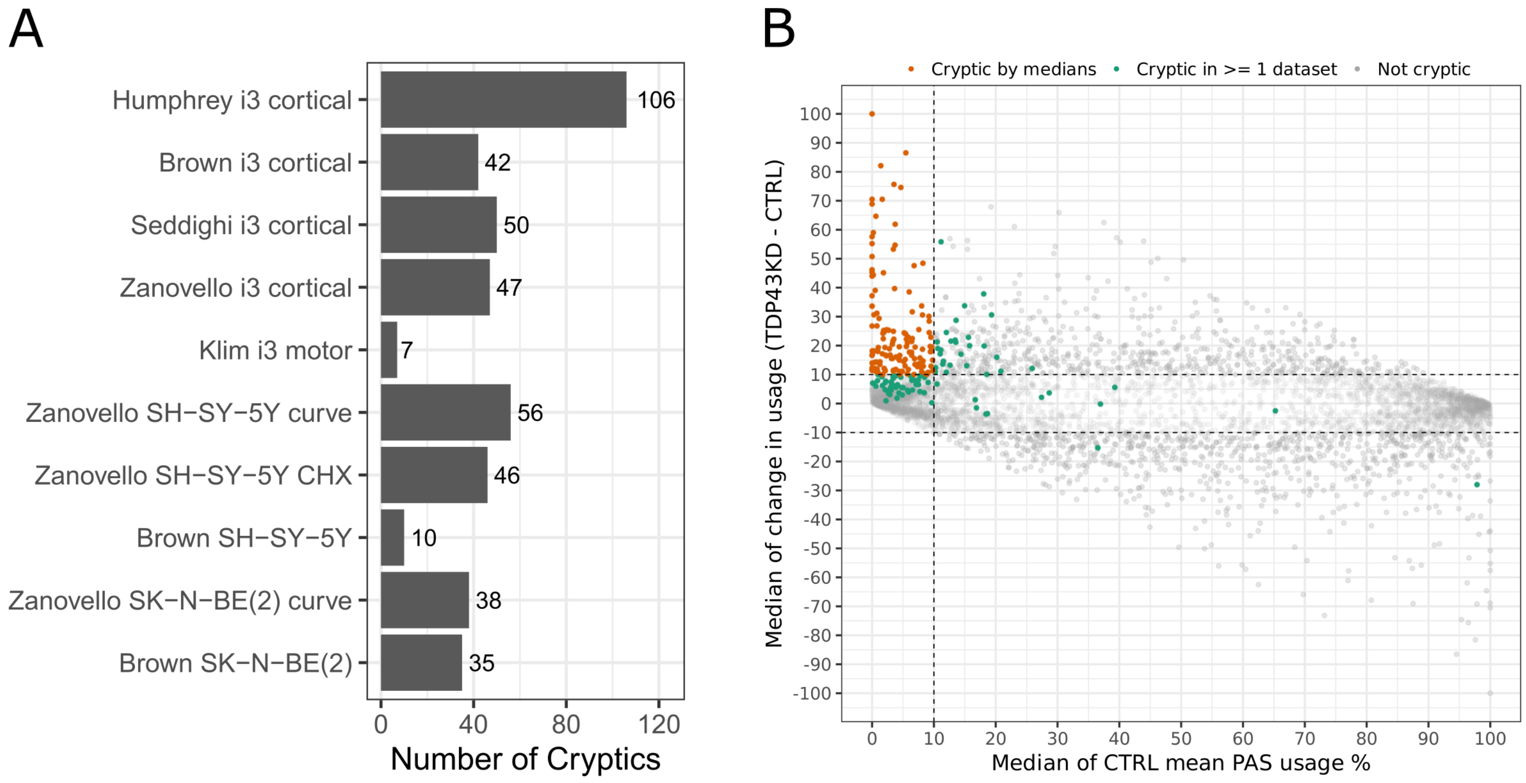

Supplementary Figure 5

Supplementary Figure 5 - Consistency of cryptic status across compendium of *in vitro* datasets

A) Bar plot of the number of cryptic APAs detected in the different *in vitro* datasets.

B) Last exons responsive to TDP-43 depletion. All points represent a last exon passing a Benjamini-Hochberg adjusted p-value < 0.05 threshold in at least one dataset. Where a last exon passes the threshold in multiple datasets, the median values across datasets are calculated to represent the basal usage (x axis) and change in usage upon TDP-43 depletion (y axis). Points that pass cryptic expression criteria in at least one dataset but pass (orange) or fail (green) the criteria when calculating the median change in usage and expression in control (CTRL) cells across datasets with an Benjamini-Hochberg adjusted p-value < 0.05 are highlighted.

A

AA-containing motifs	GUGUGA, AAUGAA, GAAUGA, UGAAUG, AUGAAU, GUGAAU, GAAUGU, UUGAAU
YA-containing motifs	AUGUGU, GUAUGU, GUGUAU, UGUGUA, UGUAUG, UGCAUG
YG-containing motifs	UGUGUG, GUGUGU, UGUGCG, UGCGUG, CGUGUG, GUGUGC
Combined motifs	All of above

B

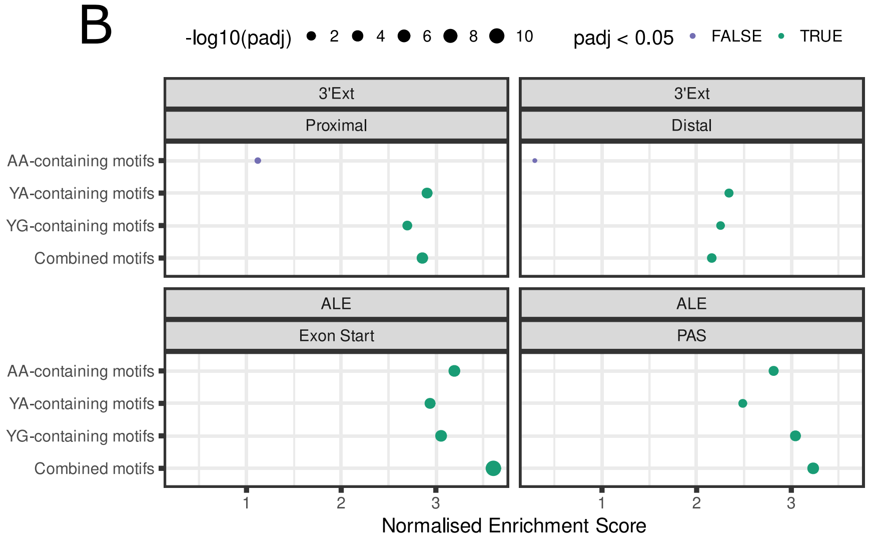

Supplementary Figure 6

Supplementary Figure 6 - Enrichment of previously defined TDP-43 binding hexamers at cryptic APA boundaries

A) Table listing previously defined TDP-43 hexamer groups[2]. ‘Y’ codes for a pyrimidine nucleotide.

B) Gene set enrichment analysis (GSEA) of enriched TDP-43 binding 6mers on de-novo enriched 6-mers around cryptic landmarks. The panels and labels correspond to regions evaluated for iCLIP binding as in Fig. 1D. The area of the points is proportional to the $-\log_{10}$ transformed adjusted p-value (adjusted with respect to all region types and motif groups), and the colour denotes whether the Benjamini-Hochberg adjusted p-value passes (green) or fails (purple) a significance threshold of < 0.05. AA-containing motifs p-values (5 d.p.): 0.43583 (3’Ext Proximal), 0.83017 (3’Ext Distal), 0.00003 (ALE Exon Start), 0.00263 (ALE PAS). YA-containing motifs: 0.00033 (3’Ext Proximal), 0.01823 (3’Ext Distal), 0.00029 (ALE Exon Start), 0.02643 (ALE PAS). YG-containing-motifs: 0.00411 (3’Ext Proximal), 0.03623 (3’Ext Distal), 0.00003 (ALE Exon Start), 0.00032 (ALE PAS). Combined motifs: 0.00008 (3’Ext Proximal), 0.00665 (3’Ext Distal), 2.68703×10^{-11} (ALE Exon Start), 0.00003 (ALE PAS).

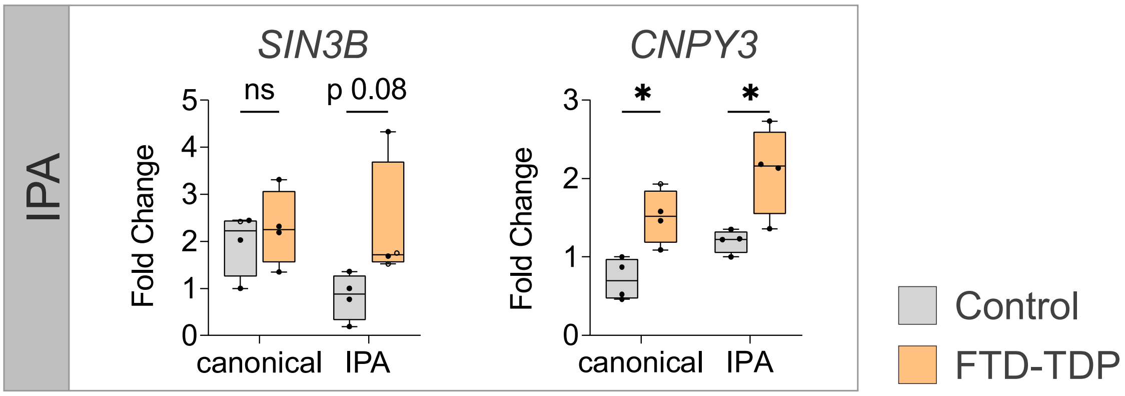

Supplementary Figure 7

Supplementary Figure 7 - 3' RACE validation of cryptic IPA events in Frontal Cortex from controls and FTD patients with TDP-43 pathology

RT-qPCR analysis after 3' RACE for the indicated IPA events in Frontal Cortex samples of control patients ("Control", n=4) and frontotemporal dementia ("FTD-TDP", n=4) cases with TDP-43 pathology. The RNA expression levels were normalized against *GAPDH* mRNA and expressed as relative fold change with respect to one control sample set to a value of 1. Data are represented as box plots (first, second and third quartile) and whiskers span from the minimum to the maximum value. Statistical analyses were performed using Student unpaired t-test (n.s. $p>0.05$, * $p<0.05$). IPA: intronic polyadenylation. *SIN3B* p = 0.568 (canonical), 0.082 (IPA). *CNPY3* p = 0.010 (canonical), 0.021 (IPA). All p-values to 3 d.p.

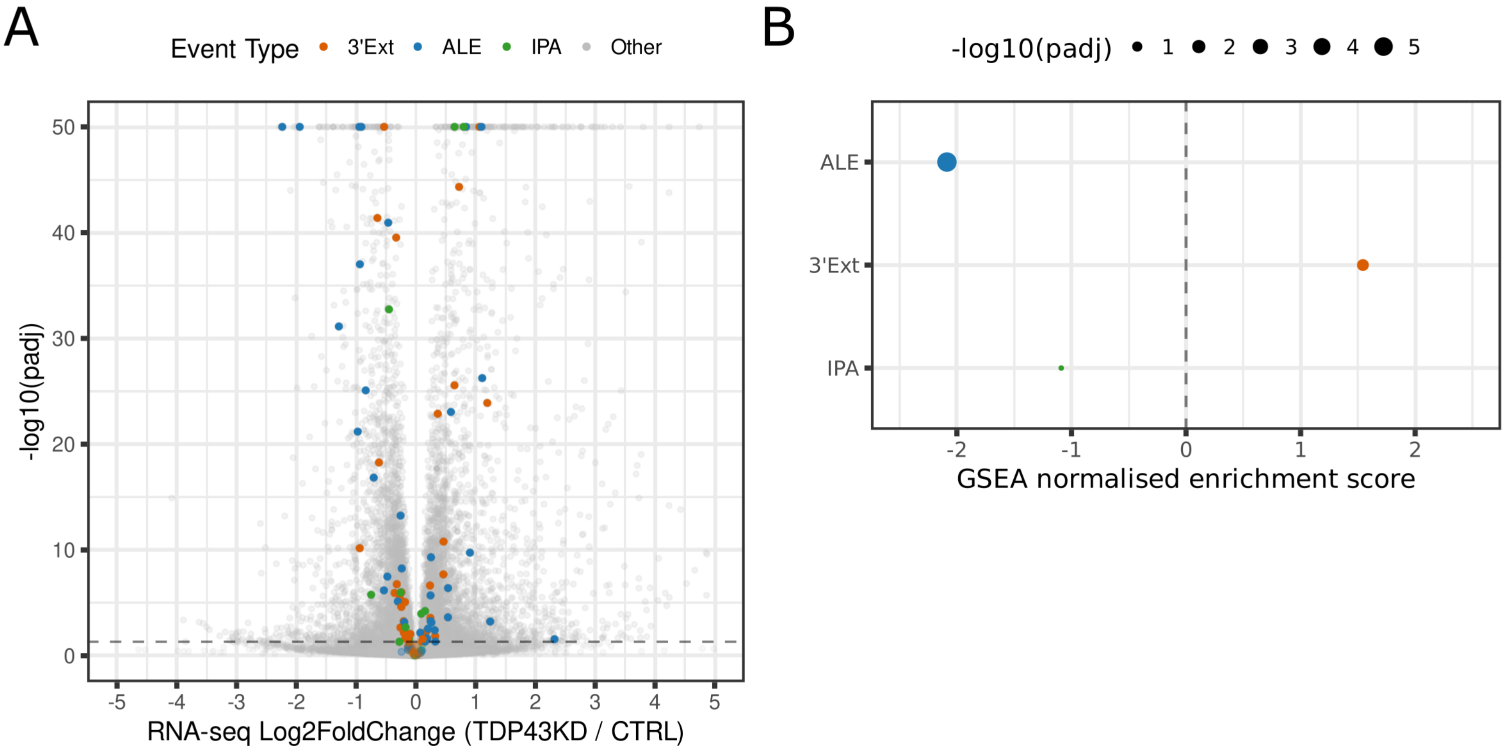

Supplementary Figure 8

Supplementary Figure 8 - Analysis of cryptic APA categories in i3Neuron RNA-seq and Ribo-seq data

A) Volcano plot of differential expression analysis of RNA-seq data between TDP-43 knockdown (TDP43KD) and control (CTRL) i3Neurons. Cryptic APA genes with significant differential expression (Benjamini-Hochberg adjusted p-value < 0.05) are highlighted in orange (3'Ext), blue (ALE) or green (IPA). Genes with a $-\log_{10}$ transformed Benjamini-Hochberg adjusted p-value greater than 50 are collapsed to 50 for visualisation purposes.

B) Gene Set Enrichment Analysis of cryptic APA categories in i3Neuron Ribo-seq differential expression fold change ranks. The area of the points is proportional to the $-\log_{10}$ transformed Benjamini-Hochberg adjusted p-value. Points are coloured according to their APA category as in A). Adjusted p-values: 2.31×10^{-6} (ALE), 0.03 (3'Ext), 0.36 (IPA).

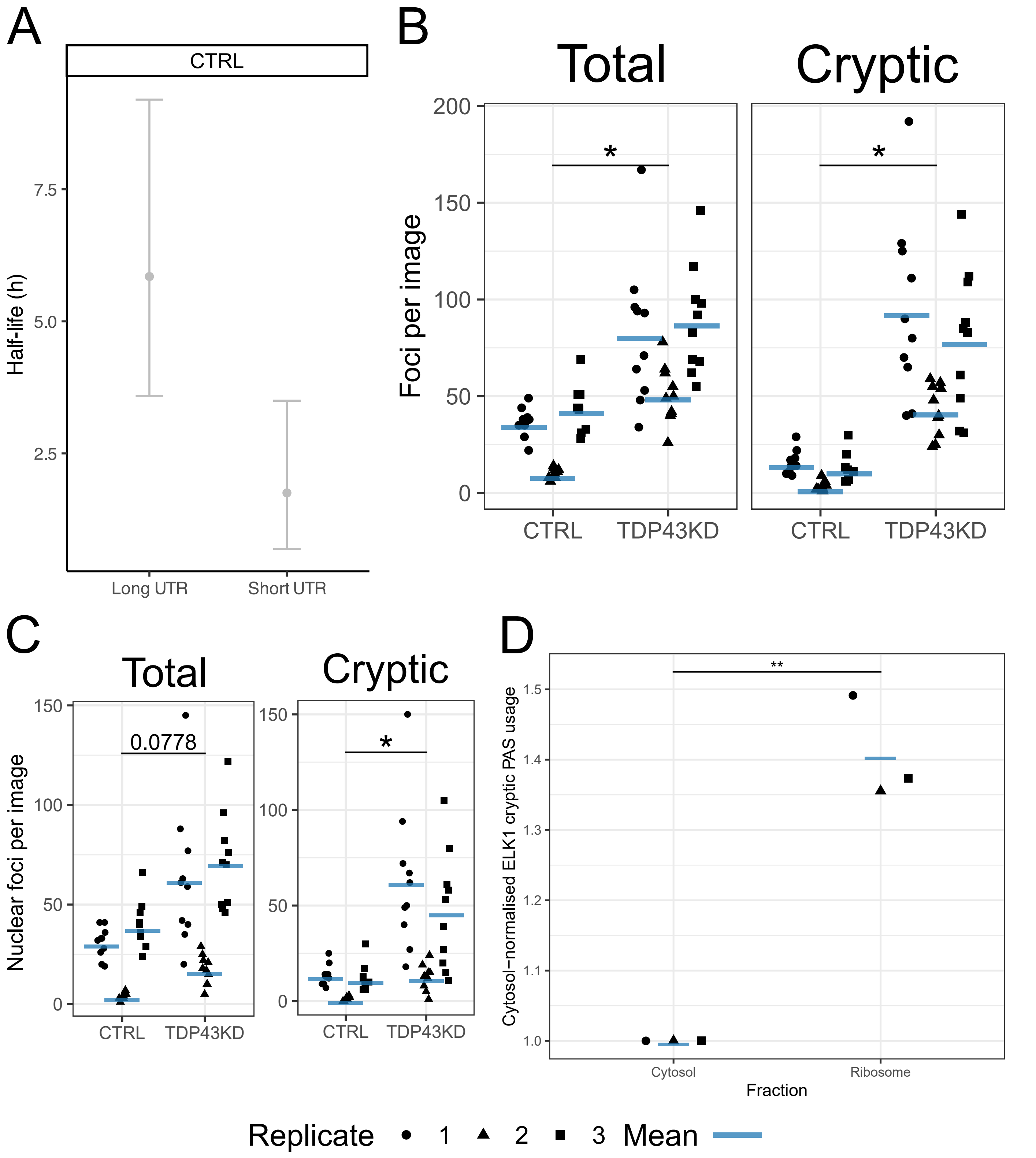

Supplementary Figure 9

Supplementary Figure 9 - *ELK1* cryptic 3'Ext isoform-specific RNA stability, localisation and ribosome association

A) Estimated half-lives for reads aligning solely to the 3'Ext-specific ('Long UTR') or annotated 3'UTR region ('Short UTR') of *ELK1* in control i3Neurons where both isoforms are detected. Half-life from estimated decay constant as the point and 95% confidence interval shown as error bars.

B) Total FISH probe signals ('Foci') for probes targeting the annotated 3'UTR region ('Total') and cryptic 3'UTR specific ('Cryptic') sequences of *ELK1* show an increase upon TDP-43 knockdown. Different shapes represent independent replicates. Individual points are images for each replicate and condition (n=10), with the mean represented as a blue horizontal bar. Statistical analysis performed using a two-sided, one-sample t-test with the means of each replicate (n = 3, * p<0.05, 'Total' p = 0.021, 'Cryptic' p = 0.005 (3 d.p.))

C) Nuclear quantification of FISH signal ('Foci') for the probes targeting the annotated 3'UTR region ('Total') and cryptic-specific 3'UTR region ('Cryptic') of *ELK1*. Different shapes represent independent replicates. Individual points are images for each replicate and condition (n=10), with the mean represented as a blue horizontal bar. Statistical analysis performed using a two-sided, one-sample t-test with the means of each replicate (n=3, * p<0.05, 'Total' p = 0.078, 'Cryptic' p = 0.028 (3 d.p.)).

D) Ratio of *ELK1* cryptic 3'Ext usage in Frac-seq data from neural progenitor cells[3]. For each replicate, PAS expression was pooled across all ribosomal fractions (monosome, light and heavy polysome), % usage was recalculated and normalised to the % usage in the cytosolic fraction. P value from two-sided, one-sample t-test on log-transformed ratios (Methods), ** p = 0.008 (3 d.p.).

Supplementary Figure References

1. Seddighi, S. *et al.* Mis-spliced transcripts generate de novo proteins in TDP-43-related ALS/FTD. *Sci. Transl. Med.* **0**, eadg7162 (2024).
2. Hallegger, M. *et al.* TDP-43 condensation properties specify its RNA-binding and regulatory repertoire. *Cell* **184**, 4680-4696.e22 (2021).
3. Ritter, A. J., Draper, J. M., Vollmers, C. & Sanford, J. R. Long-read subcellular fractionation and sequencing reveals the translational fate of full-length mRNA isoforms during neuronal differentiation. *Genome Res.* **34**, 2000–2011 (2024).
