# Supplementary material for: TDP-43 loss induces cryptic polyadenylation in ALS/FTD
Source: Nat Neurosci. 2025 Oct 21;28(11):2190–200. doi: 10.1038/s41593-025-02050-w (PMC12586162; doi:10.1038/s41593-025-02050-w)
Supplement: Supplementary file 2 — Reporting Summary [file 41593_2025_2050_MOESM2_ESM.pdf]

nature portfolio

Corresponding author(s): Maria Secrier, Pietro Fratta

Last updated by author(s): May 27, 2025

Reporting Summary

Nature Portfolio wishes to improve the reproducibility of the work that we publish. This form provides structure for consistency and transparency in reporting. For further information on Nature Portfolio policies, see our Editorial Policies and the Editorial Policy Checklist.

Statistics

For all statistical analyses, confirm that the following items are present in the figure legend, table legend, main text, or Methods section.

n/a	Confirmed	
☐	☒	The exact sample size (*n*) for each experimental group/condition, given as a discrete number and unit of measurement
☐	☒	A statement on whether measurements were taken from distinct samples or whether the same sample was measured repeatedly
☐	☒	The statistical test(s) used AND whether they are one- or two-sided<br>*Only common tests should be described solely by name; describe more complex techniques in the Methods section.*
☐	☒	A description of all covariates tested
☐	☒	A description of any assumptions or corrections, such as tests of normality and adjustment for multiple comparisons
☐	☒	A full description of the statistical parameters including central tendency (e.g. means) or other basic estimates (e.g. regression coefficient) AND variation (e.g. standard deviation) or associated estimates of uncertainty (e.g. confidence intervals)
☐	☒	For null hypothesis testing, the test statistic (e.g. *F*, *t*, *r*) with confidence intervals, effect sizes, degrees of freedom and *P* value noted<br>*Give P values as exact values whenever suitable.*
☐	☒	For Bayesian analysis, information on the choice of priors and Markov chain Monte Carlo settings
☒	☐	For hierarchical and complex designs, identification of the appropriate level for tests and full reporting of outcomes
☒	☐	Estimates of effect sizes (e.g. Cohen's *d*, Pearson's *r*), indicating how they were calculated

Our web collection on statistics for biologists contains articles on many of the points above.

Software and code

Policy information about availability of computer code

Data collection	No specialised software was used for data collection
Data analysis	The following software and annotation versions were used for the preprocessing of the 'Humphrey i3 Cortical' dataset:<br>Trimmomatic 0.36<br>STAR 2.7.2a<br>GRCh38 genome build<br>Gencode v30 transcript annotations<br><br>The pipeline is deposited on GitHub at https://github.com/CommonMindConsortium/RAPiD-nf/<br><br>For processing of all other 'standard' RNA-seq datasets, the following software and annotation files were used:<br>fastp 0.20.1<br>STAR 2.7.8a<br>GRCh38 genome build<br>Gencode v40 transcript annotations<br><br>The pipeline is deposited on GitHub and Zenodo (https://github.com/frattalab/rna_seq_snakemake , https://doi.org/10.5281/zenodo.15463283)<br><br>For SLAM-seq processing and analysis:<br>fastp 0.20.1

STAR v.2.7.0f
GRCh38 genome build
Gencode v40 annotations
GRAND-SLAM 2.0.7b
fastq2EZbakR 0.2.0
EZbakR 0.0.0.9000
grandR 0.2.2

For the PAPA pipeline:
StringTie 2.1.7
Gffcompare 0.11.2
PolyASite 2.0
Gffread 0.12.1
Salmon 1.5.2
Tximport v1.26.0
DEXSeq v1.44.0
R 4.2.2
Snakemake 6.7.0
PyRanges 0.0.115
Pyfaidx 0.6.2
Python 3.8.10

Version 0.2.0 was used for the manuscript. The pipeline is available on GitHub and is archived at Zenodo (https://github.com/frattalab/PAPA , https://doi.org/10.5281/zenodo.15210362).

The poly(A)-tail containing read (PATR) extraction and clustering pipeline ('bulk_polyatail_reads'):
Nullranges 1.8.0
Cobalt 4.5.5
Snakemake 7.32.4
Python 3.10.13
pyranges 0.0.129
Pysam 0.22.0
Pandas 2.1.4
Numpy 1.26.3
Pyarrow 15.0.0
Fastparquet 2024.2.0

Version 0.1.0 was used for the manuscript. The pipeline is available on GitHub and is archived at Zenodo (https://github.com/SamBryce-Smith/bulk_polyatail_reads , https://doi.org/10.5281/zenodo.15210306).

DaPars2 comparison:
NCBI RefSeq v110 transcripts
APAeval commit ID d7831b6 (https://github.com/iRNA-COSI/APAeval)
DaPars2 commit ID 23d89d1 (https://github.com/3UTR/DaPars2)

ELK1 3'UTR reporter:
minimap 2.28
python 3.6.13 (general), 3.9.19 (SpliceAI)
pysam 0.21.0
SpliceAI 1.3.1
keras 2.12.0
dnaio 0.7.1

Version 1.0 was used for the manuscript. The analysis code is available on GitHub and is archived at Zenodo (https://github.com/MaxChien1996/replace_UG_in_first_800_bp_of_ELK1_extended_3_prime_UTR , https://doi.org/10.5281/zenodo.15413618)

the 'salmon' and 'feature_counts' subpipelines:
salmon 1.8.0
featureCounts v.2.0.1

The pipelines are deposited at GitHub and Zenodo (https://github.com/frattalab/rna_seq_single_steps , https://doi.org/10.5281/zenodo.15210438)

The splice-junction read quantification pipeline:
bedops 2.4.39
bedtools 2.30.0
python 3.8.6

v0.1.0 was used in the manuscript. The code is deposited on GitHub and Zenodo (https://github.com/SamBryce-Smith/bedops_parse_star_junctions , https://doi.org/10.5281/zenodo.15209898).

The remaining custom analysis code is deposited in the 'tdp43-apa' GitHub repository and is archived at Zenodo (https://github.com/frattalab/tdp43-apa , https://doi.org/10.5281/zenodo.15210472). This code uses the following software:
R 4.3.2
ggplot2 3.4.4
ggpubr 0.6.0

ggprism 1.0.4
ggrepel 0.94
tidyverse 2.0.0
writexl 1.4.2
data.table 1.14
Python 3.10.11
PyRanges 0.0.127
pandas 2.0.2
numpy 1.23
snakemake 7.26.0
bedtools 2.31.0
PEKA (forked copy commit ID f934395, 'output_mods' branch at https://github.com/SamBryce-Smith/peka)
cv_coverage 1.1.0
DESeq2 1.38.3
fgsea 1.24.0
MAJIQ 2.4
nullranges 1.8.0
cobalt 4.5.5
ImageJ v1.54f was used for fluorescent in-situ hybridisation image analysis and foci quantification.

For manuscripts utilizing custom algorithms or software that are central to the research but not yet described in published literature, software must be made available to editors and reviewers. We strongly encourage code deposition in a community repository (e.g. GitHub). See the Nature Portfolio guidelines for submitting code & software for further information.

Data

Policy information about availability of data

All manuscripts must include a data availability statement. This statement should provide the following information, where applicable:

- Accession codes, unique identifiers, or web links for publicly available datasets
- A description of any restrictions on data availability
- For clinical datasets or third party data, please ensure that the statement adheres to our policy

This study analyses existing and newly generated datasets. All existing datasets are publicly available from the accessions reported below. 'Brown' i3Neuron, SH-SY5Y and SK-N-BE(2) datasets are available through the European Nucleotide Archive (ENA) under accession PRJEB42763. The SH-SY5Y TDP-43 iCLIP data is available at ENA under accession PRJEB49480 or ArrayExpress under accession E-MTAB-11243. 'Seddighi' i3Neuron RNA-seq, i3Neuron Nanopore direct RNA-seq and i3Neuron Ribo-seq data can be accessed at Alzheimer's Disease Workbench (ADWB): https://fair.addi.ad-datainitiative.org/#/data/datasets/mis_spliced_transcripts_generate_de_novo_proteins_in_tdp_43_related_als_ftd_00005. The HeLa TDP-43 Knockout (GSE136366), FACS-sorted frontal cortex neuronal nuclei (GSE126543) and the 'Klim' iPSC-derived motor neurons (GSE12156) can be accessed via Gene Expression Omnibus (GEO). Raw ChIP-seq data for ELK1 (GSM608163, GSM935326) and ELK4 (GSM608161, GSM608162, GSM935351) in HeLa cells can also be accessed through GEO or in processed format as used in this study via ChIP-atlas (https://chip-atlas.org/). The short-read neural progenitor cell Frac-seq data was downloaded from the GEO at accession number GSE244655.

RNA-seq data generated by the NYGC ALS Consortium and used in this study can be accessed through the GEO database (GSE137810, GSE124439, GSE116622, GSE153960). To request immediate access to new and ongoing data generated by the NYGC ALS Consortium and for samples provided through the Target ALS Postmortem Core, complete a genetic data request form at ALSData@nygenome.org.

All sequencing datasets generated in this study have been deposited at the GEO database: 'Zanovello i3Neuron' (GSE296710), 'Humphrey i3Neuron' (GSE296714), 'Zanovello SH-SY5Y CHX' (GSE296713), 'Zanovello SH-SY5Y curve' (GSE296712), 'Zanovello SK-N-BE(2) curve' (GSE296711) and i3Neuron SLAM-seq (GSE296716). An archive of minimal processed data required to reproduce analysis and figures presented in this manuscript is available from Zenodo (https://doi.org/10.5281/zenodo.15538002).

The following genome sequence and transcriptome annotation versions were used:
GRCh38 genome build - https://www.ncbi.nlm.nih.gov/datasets/genome/GCF_000001405.26/
Gencode v30 (Humphrey i3 Cortical, v34 (SLAM-seq) and v40 (all others) transcript annotations - https://ftp.ebi.ac.uk/pub/databases/gencode/Gencode_human/
PolyASite 2.0 - https://www.polyasite.unibas.ch/download/atlas/2.0/GRCh38.96/atlas.clusters.2.0.GRCh38.96.bed.gz

Research involving human participants, their data, or biological material

Policy information about studies with human participants or human data. See also policy information about sex, gender (identity/presentation), and sexual orientation and race, ethnicity and racism.

Reporting on sex and gender	Sex was collected for all individuals in the NYGC ALS Consortium dataset and was verified using the RNA-seq expression of the sex-specific marker genes XIST and UTY. Analysis of selective expression in post-mortem tissue (Fig 2) was performed without considering sex, because the analysis discriminates between samples with and without inferred TDP-43 pathology which is not determined by sex.
Reporting on race, ethnicity, or other socially relevant groupings	None used because no socially constructed categorization variables were recorded in the provided NYGC ALS Consortium metadata.
Population characteristics	1682 tissue samples from 446 unique participants (203 female).<br>Control – 104 individuals (50 female), median age 65 (interquartile range 19.5)

	ALS – 279 individuals (127 female), median age 66 (interquartile range 12)<br>FTD – 63 individuals (26 female), median age 67 (interquartile range 10)
Recruitment	In NYGC ALS Consortium the recruitment and contribution of postmortem samples and clinical information was performed by Consortium members using their recruitment criteria and strategy
Ethics oversight	The NYGC ALS Consortium samples presented in this work were acquired through various institutional review board (IRB) protocols from member sites and the Target ALS postmortem tissue core and transferred to the NYGC in accordance with all applicable foreign, domestic, federal, state, and local laws and regulations for processing, sequencing, and analysis. The Biomedical Research Alliance of New York (BRANY) IRB serves as the central ethics oversight body for NYGC ALS Consortium. Ethical approval was given. Informed consent has been obtained from all participants.

Note that full information on the approval of the study protocol must also be provided in the manuscript.

Field-specific reporting

Please select the one below that is the best fit for your research. If you are not sure, read the appropriate sections before making your selection.

☒ Life sciences ☐ Behavioural & social sciences ☐ Ecological, evolutionary & environmental sciences

For a reference copy of the document with all sections, see nature.com/documents/nr-reporting-summary-flat.pdf

Life sciences study design

All studies must disclose on these points even when the disclosure is negative.

Sample size	NYGC ALS consortium sample size was not pre-determined as data collection is still ongoing. Sample size was determined by the number of available RNA-seq samples at time of analysis, which corresponded to a subset of the 2023-02-21 data freeze. Overall sample sizes are reported in 'Population Characteristics' section above and split by TDP-43 pathology status in Supplementary Table 4.<br>Sample sizes for novel cell-line RNA-seq datasets and experimental validation were not determined by formal power analysis. Instead, sample sizes were determined based on prior studies similarly aiming to identify novel isoforms, perform targeted validation and assess their downstream effects on RNA and protein expression. Examples of such prior studies include “TDP-43 loss and ALS-risk SNPs drive mis-splicing and depletion of UNC13A”.<br>Sample sizes for novel RNA-seq experiments (CTRL = Control, KD = TDP-43 knockdown):<br>'Zanovello SH-SY-5Y CHX' - 4 CTRL, 4 KD<br>'Zanovello SH-SY-5Y Curve' - 3 CTRL, 3 KD<br>'Zanovello SK-N-BE(2) Curve' - 3 CTRL, 3 KD<br>'Zanovello i3 Cortical' - 4 CTRL, 4 KD<br>'Seddighi i3 Cortical' - 12 CTRL, 6 KD<br>'Humphrey i3 Cortical' - 6 CTRL, 6 KD<br>Sample sizes for novel and previously published RNA-seq datasets ('Brown SH-SY5Y', 'Brown SK-N-BE(2)', 'Brown i3 Cortical', 'Klim i3 Motor') are further described in Supplementary Table 1.<br><br>Sample sizes for non-RNAseq experiments were not determined using formal statistical methods. Sample sizes for 3'RACE-based cryptic APA validation in post-mortem tissue was determined by sample availability at the time of analysis. For all other targeted experimental assays, sample sizes were based on technical feasibility and previous studies investigating changes induced by novel RNA isoforms, such as “TDP-43 loss and ALS-risk SNPs drive mis-splicing and depletion of UNC13A”. Sample sizes are as follows:<br>- Halo-i3Neuron ELK1 Western blot (Fig. 3C) - 4 CTRL, 4 KD<br>- i3Neuron 3'RACE validation (Extended Data Fig. 1) - 4 CTRL, 4 KD<br>- ELK1 3'UTR reporter library (Fig 1H, Extended Data Fig 2)) - n = 3 for each variant and experimental condition (doxycycline concentration)<br>- Frontal cortex tissue cryptic APA 3'RACE (Fig. 2B, Supplementary Fig 7) - 4 CTRL, 4 FTD-TDP<br>- ELK1 FISH in i3Neurons (Fig. 3G, Supplementary Fig. 9B,C) - 3 CTRL, 3 KD<br>- Sub-cellular fractionation in SH-SY5Y cells (Fig. 3H, Extended Data Fig 4) - 3 CTRL, 3 KD
Data exclusions	None reported.
Replication	All RNA-seq, SLAM-seq and Ribo-seq experiments involved multiple biological replicates in each condition, and statistical analyses that model variability between replicates were used to model average effect sizes and to prioritise targets with differences between experimental conditions. ELK1 protein upregulation was reported in i3Neuron models with different mechanisms and developmental timing of TDP-43 loss, and reproduced across 4 independent differentiations (Fig 3C).<br>ELK1 cryptic 3'Ext RNA upregulation in the extra-nuclear compartment was confirmed by independent assays in different cellular models (FISH = i3Neurons, biochemical fractionation combined with 3'RACE = SH-SY5Y). Each assay was performed using independent differentiations and the relative patterns between the experimental condition were consistent across all replicates. All attempts at replication were successful.
Randomization	The majority of analyses in this study was carried out in cell lines, which do not require randomization due to their inherent homogeneity. The omics data were generated in a high throughput manner and intended for generic analyses of changes in context of TDP-43 depletion. Where novel targets were highlighted via transcriptome-wide analysis, orthogonal biochemical assays were performed to validate these initial observations.
Blinding	Fluorescent in-situ hybridisation images were analysed blinded to TDP-43 depletion status. All other investigations were performed unblinded to experimental condition or disease status. In these cases, blinding is not applicable because the data generation/quantification are automated procedures that do not involve subjective interpretation.

Reporting for specific materials, systems and methods

We require information from authors about some types of materials, experimental systems and methods used in many studies. Here, indicate whether each material, system or method listed is relevant to your study. If you are not sure if a list item applies to your research, read the appropriate section before selecting a response.

Materials & experimental systems

n/a	Involved in the study
☐	☒ Antibodies
☐	☒ Eukaryotic cell lines
☒	☐ Palaeontology and archaeology
☒	☐ Animals and other organisms
☒	☐ Clinical data
☒	☐ Dual use research of concern
☒	☐ Plants

Methods

n/a	Involved in the study
☒	☐ ChIP-seq
☒	☐ Flow cytometry
☒	☐ MRI-based neuroimaging

Antibodies

Antibodies used

anti-ELK1 (Abcam ab32106) 1:500
anti-TDP-43 (Abcam, ab104223) 1:2000
anti-tubulin (Sigma-Aldrich, MAB1637) 1:5000
anti-mouse HRP (BioRad, 1706516) 1:10000
anti-rabbit HRP (BioRad, 1706515) 1:10000)

Validation

anti-ELK1 (Abcam ab32106) has been validated in ELK1 knockout HeLa cells and cited in 46 publications
anti-TDP-43 (Abcam, ab104223) has been validated in TDP-43 knockout HAP1 cells and cited in 18 publications
anti-tubulin (Sigma-Aldrich, MAB1637) has been validated in mouse brain tissue lysates (positive control) and non-neuronal tissue (negative control. Cited in 413 publications.
anti-mouse HRP (BioRad, 1706516) has been used in > 1000 citations.
anti-rabbit HRP (BioRad, 1706515) according to the manufacturer's website has been double-affinity purified with human IgG adsorbed.

Eukaryotic cell lines

Policy information about cell lines and Sex and Gender in Research

Cell line source(s)

All iPS-derived cortical neurons (i3Neurons) used in this study are from the WTC11 line, which was derived from a healthy human male participant. All policies of the NIH Intramural Research Program for the registration and use of this iPS cell line were followed. SH-SY5Y cells were obtained from ATCC. SK-N-BE(2) cells were obtained from the International Centre for Genetic Engineering and Biotechnology in Trieste, Italy.

Authentication

WTC11 iPS cell line was validated to have a normal male karyotype. SK-N-BE(2) and SH-SY5Y cell lines were validated by Cell Services at The Francis Crick Institute.

Mycoplasma contamination

WTC11 iPS cell line was confirmed to be mycoplasma free based on the Lonza MycoAlert mycoplasma testing kit. SH-SY-5Y and SK-N-BE(2) cells were confirmed to be mycoplasma free using the PHOENIXDX® MYCOPLASMA MIX qPCR kit by Procomcure Biotech

Commonly misidentified lines
(See ICLAC register)

No commonly misidentified cell lines were used in this study.

Plants

Seed stocks

Report on the source of all seed stocks or other plant material used. If applicable, state the seed stock centre and catalogue number. If plant specimens were collected from the field, describe the collection location, date and sampling procedures.

Novel plant genotypes

Describe the methods by which all novel plant genotypes were produced. This includes those generated by transgenic approaches, gene editing, chemical/radiation-based mutagenesis and hybridization. For transgenic lines, describe the transformation method, the number of independent lines analyzed and the generation upon which experiments were performed. For gene-edited lines, describe the editor used, the endogenous sequence targeted for editing, the targeting guide RNA sequence (if applicable) and how the editor was applied.

Authentication

Describe any authentication procedures for each seed stock used or novel genotype generated. Describe any experiments used to assess the effect of a mutation and, where applicable, how potential secondary effects (e.g. second site T-DNA insertions, mosiacism, off-target gene editing) were examined.
