# Supplementary figures and images for: TDP-43 loss induces cryptic polyadenylation in ALS/FTD
Source: Nat Neurosci. 2025 Oct 21;28(11):2190–200. doi: 10.1038/s41593-025-02050-w (PMC12586162; doi:10.1038/s41593-025-02050-w)

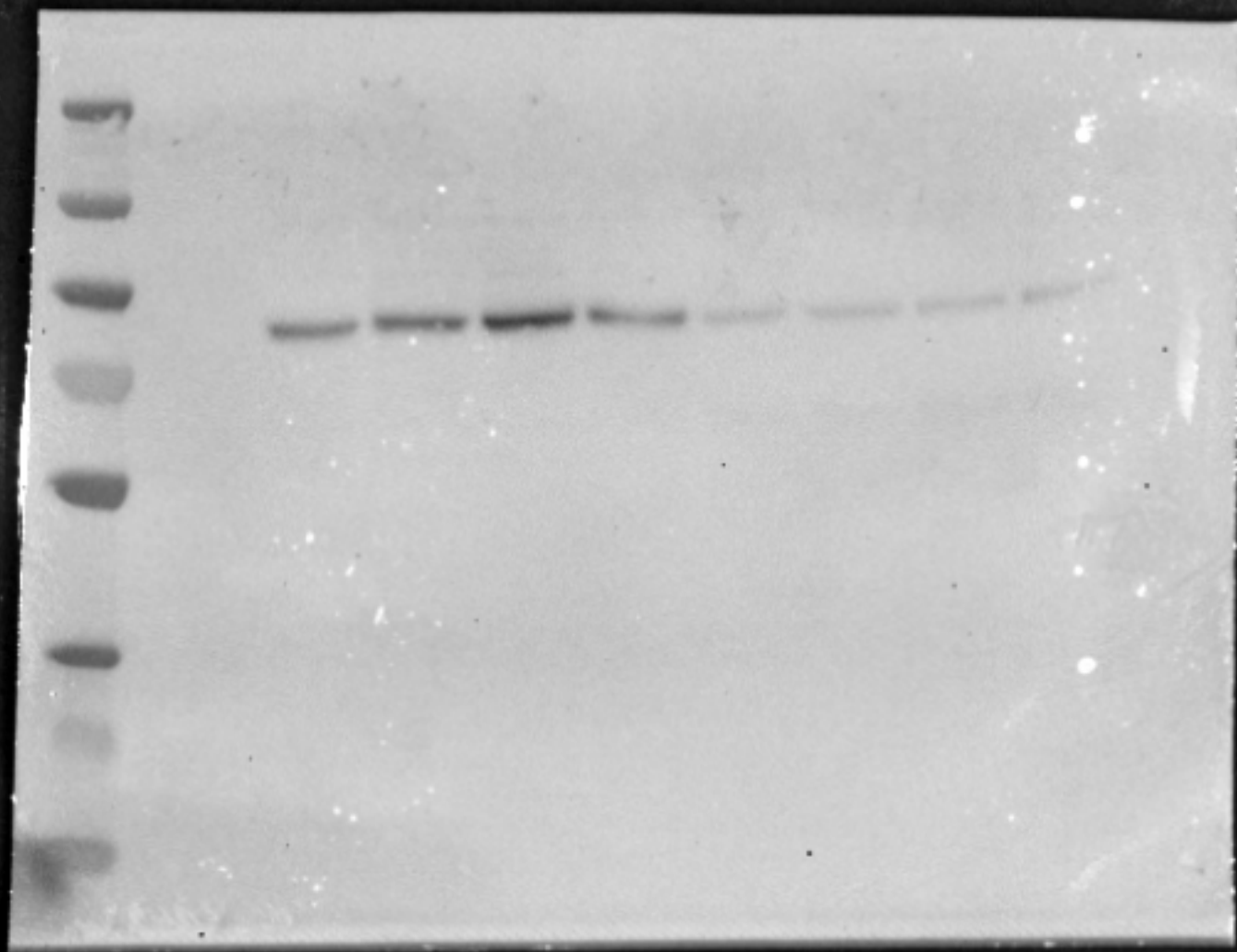

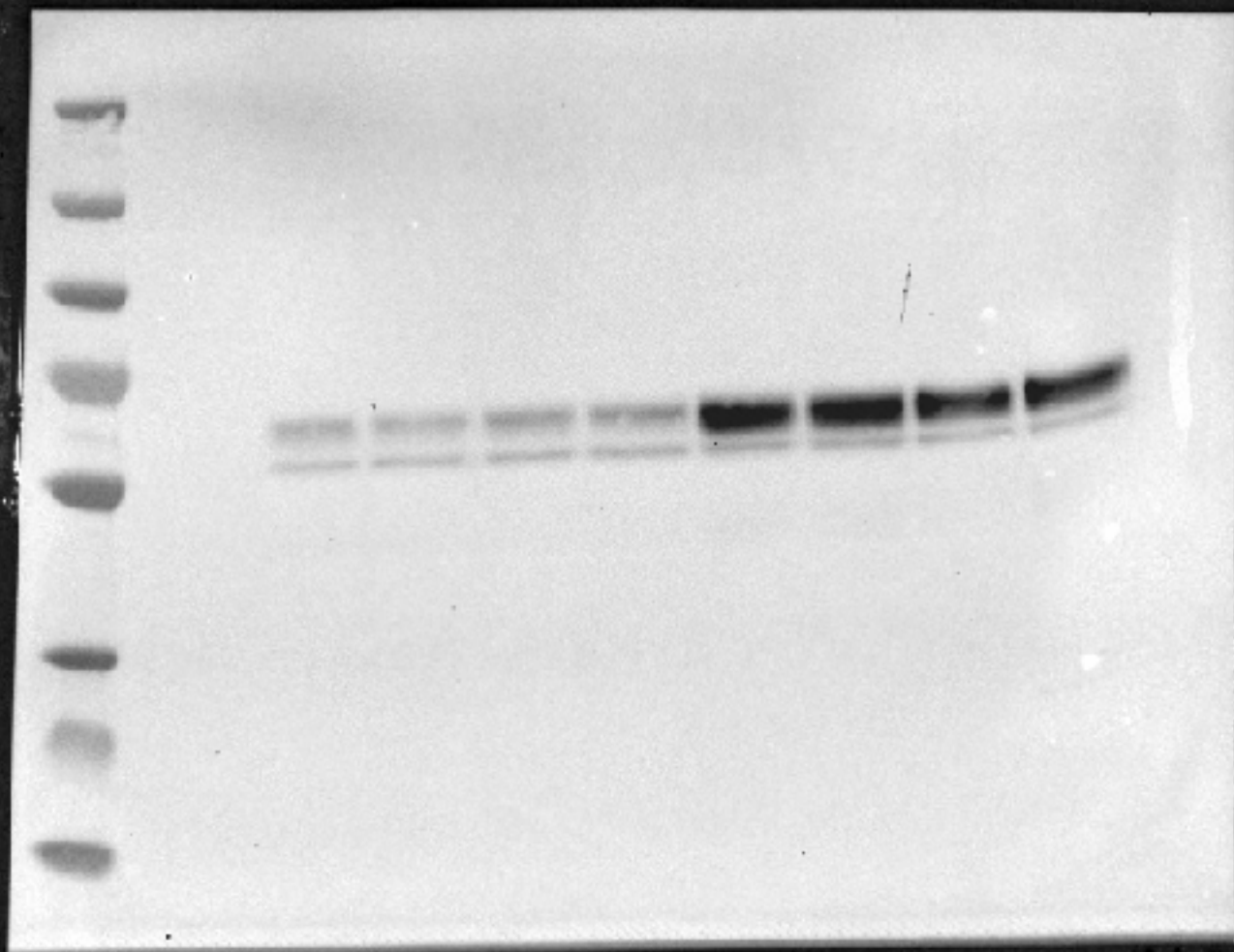

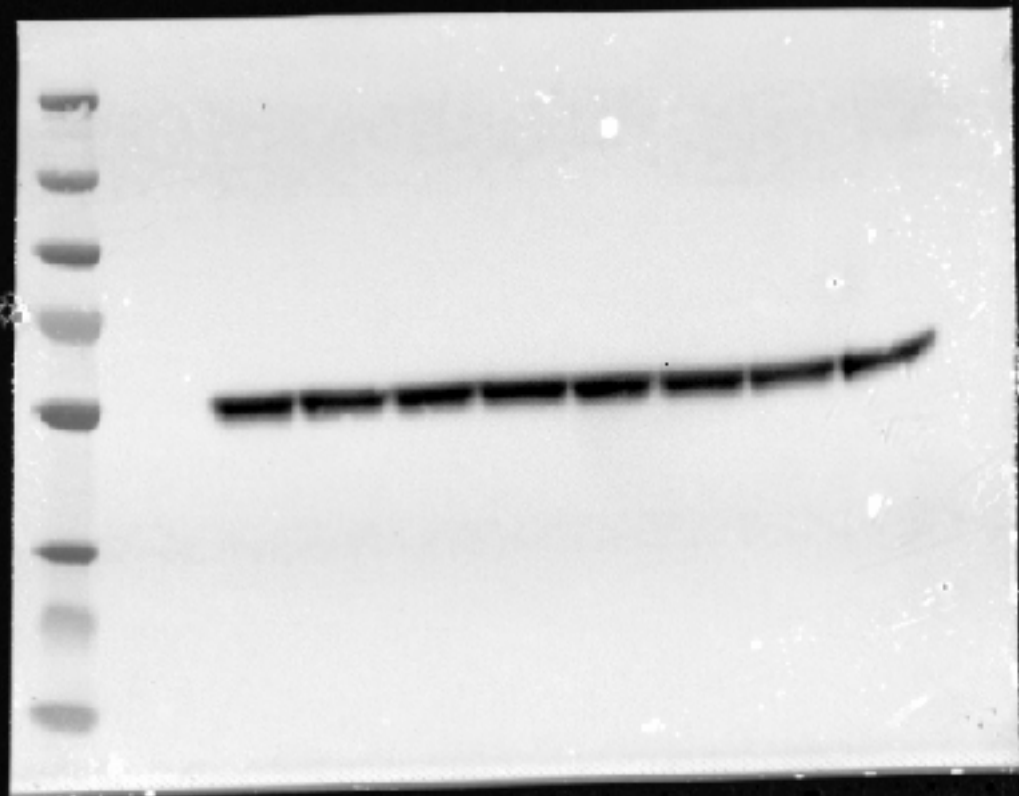

Supplement: Supplementary file 6 — Unprocessed, uncropped western blots for Fig. 3c (page 1: Halo-tagged TDP-43; page 2: ELK1; page 3: Tubulin) [file 41593_2025_2050_MOESM6_ESM.pdf]

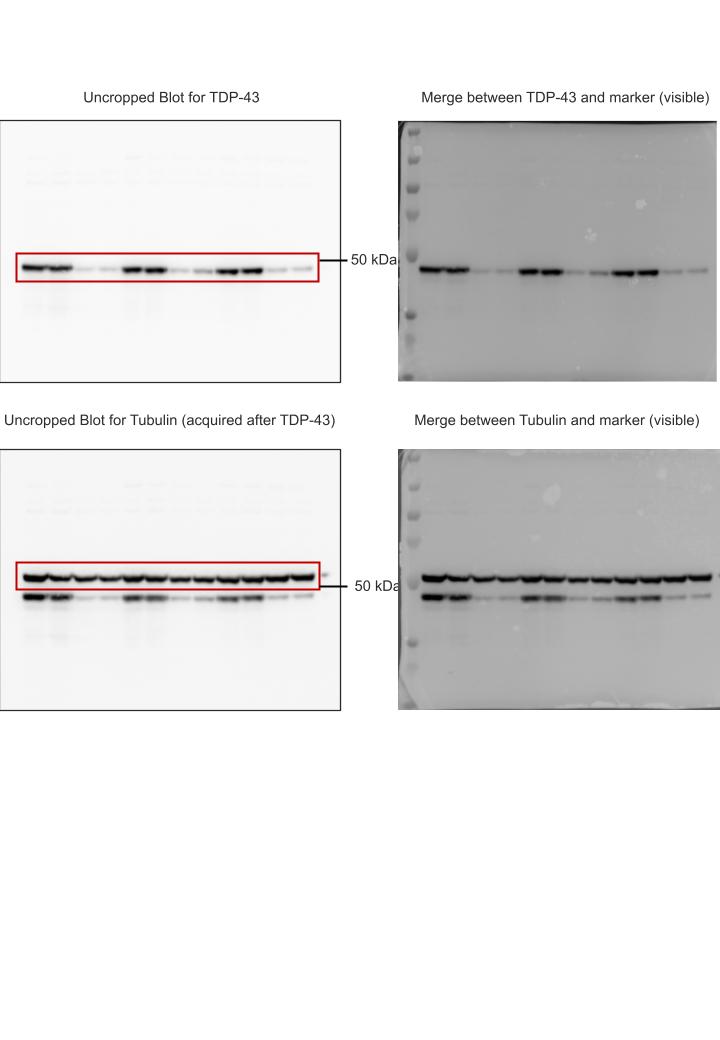

Supplement: Supplementary file 9 — Unprocessed, uncropped western blots (Extended Data Fig. 4a) [file 41593_2025_2050_MOESM9_ESM.jpg]
